# Genome-wide uniformity of human ‘open’ pre-initiation complexes

**DOI:** 10.1101/gr.210955.116

**Published:** 2017-01

**Authors:** William K.M. Lai, B. Franklin Pugh

**Affiliations:** Center for Eukaryotic Gene Regulation, Department of Biochemistry and Molecular Biology, The Pennsylvania State University, University Park, Pennsylvania 16802, USA

## Abstract

Transcription of protein-coding and noncoding DNA occurs pervasively throughout the mammalian genome. Their sites of initiation are generally inferred from transcript 5′ ends and are thought to be either locally dispersed or focused. How these two modes of initiation relate is unclear. Here, we apply permanganate treatment and chromatin immunoprecipitation (PIP-seq) of initiation factors to identify the precise location of melted DNA separately associated with the preinitiation complex (PIC) and the adjacent paused complex (PC). This approach revealed the two known modes of transcription initiation. However, in contrast to prevailing views, they co-occurred within the same promoter region: initiation originating from a focused PIC, and broad nucleosome-linked initiation. PIP-seq allowed transcriptional orientation of Pol II to be determined, which may be useful near promoters where sufficient sense/anti-sense transcript mapping information is lacking. PIP-seq detected divergently oriented Pol II at both coding and noncoding promoters, as well as at enhancers. Their occupancy levels were not necessarily coupled in the two orientations. DNA sequence and shape analysis of initiation complex sites suggest that both sequence and shape contribute to specificity, but in a context-restricted manner. That is, initiation sites have the locally “best” initiator (INR) sequence and/or shape. These findings reveal a common core to pervasive Pol II initiation throughout the human genome.

The mammalian transcription machinery assembles into a preinitiation complex (PIC) consisting of general transcription factors such as TFIIB and RNA polymerase II (Pol II) and strand-separated or open DNA (Kostrewa et al. 2009; He et al. 2013; Sainsbury et al. 2015; Bernecky et al. 2016; Louder et al. 2016). Once Pol II initiates transcription, it then forms a paused complex (PC) 20–60 bp downstream at most genes (Core et al. 2008; Adelman and Lis 2012). As such, pausing appears to be a rate-limiting step in transcription once Pol II has been recruited to promoters (Ptashne and Gann 1997; Kwak et al. 2013). Since little Pol II is detected over core promoters where general transcription factors are found, it has been widely assumed that PICs rapidly initiate and move into a paused state, leaving the general transcription factors at the promoter (Kwak et al. 2013; Jonkers and Lis 2015) or allowing them to dissociate (Zawel et al. 1995). It is also possible that the PIC and PC are sterically incompatible at promoters.

Despite the high resolution of certain genome-wide assays (Mahony and Pugh 2015), their spatial resolution may be insufficient to distinguish PICs from PCs (Core et al. 2014). RNA-based assays (e.g., CAGE, Start-seq, CapSeq) and run-on assays (e.g., GRO-cap) define the precise locations of TSSs and paused polymerases (Shiraki et al. 2003; Nechaev et al. 2010; Gu et al. 2012; The ENCODE Project Consortium 2012; Core et al. 2014). However, since these assays involve read-outs requiring more than ∼20 nucleotides (nt) of RNA for unique mappability to the genome, they do not report on pre- and early-initiation events that involve smaller RNA lengths. ChIP-exo reports with high precision the genomic locations of formaldehyde-mediated protein–DNA crosslinks (Rhee and Pugh 2012a). However, the multitude of crosslinks that overlap the ∼70 bp covered by both PICs and PCs potentially limit their resolution (Rhee and Pugh 2012b). Nevertheless, the observation that the peak of Pol II crosslinking in human cells is coincident with where Pol II pauses indicates that PICs may be relatively short-lived (Core et al. 2014), and it is unclear whether they have sufficient kinetic stability to be regulated or even detected.

To spatially resolve mammalian PICs and PCs, we sought a high-resolution assay that could specify the genomic position of the Pol II active site, regardless of whether or not RNA is synthesized. We turned to the well-established permanganate reactivity of open DNA that resides in the Pol II active site (Giardina et al. 1992). While this assay has been described on a genomic scale in *Drosophila* (Li et al. 2013), a comprehensive examination of potential nucleotide biases in the assay and its ability to detect PICs at mRNA genes was not reported. Additionally, open complexes have not been examined in human cells, which, unlike *Drosophila*, possess prevalent divergent transcription (Core et al. 2008, 2012). Distinguishing PICs from nearby PCs depends not only on the inherent positional resolution of the data but also on the accuracy of measured TSS locations and the extent to which they are focused rather than dispersed. Focused promoters utilize a single major TSS, whereas dispersed promoters display a multitude of initiation sites (Juven-Gershon et al. 2008; Juven-Gershon and Kadonaga 2010; Rach et al. 2011; Haberle et al. 2014). While they are described as separate classes (Carninci et al. 2006; Lenhard et al. 2012), dispersed promoters remain ill defined, and it is not clear whether they are physically distinct from focused promoters.

High-resolution crystal structures of TFIIB within biochemically assembled PICs indicate that TFIIB innervates into the Pol II active site that contains open DNA (He et al. 2013; Barnes et al. 2015; Sainsbury et al. 2015). These structures have enabled the assignment of points of formaldehyde-induced TFIIB–DNA and Pol II–DNA crosslinks in the PICs of budding yeast (Rhee and Pugh 2012b). Since yeast polymerases do not pause just downstream from the TSS like in metazoan cells, relatively stable PICs have been detected. However, even these PICs rapidly move into elongating complexes (Jeronimo and Robert 2014; Wong et al. 2014). The presence of TFIIB within the PIC active site suggests that experimentally coupling the single-nucleotide resolution of permanganate reactivity in open DNA with ChIP-seq (which we refer to here as PIP-seq) for TFIIB may allow the detection of the PIC. Since TFIIB is expected to be displaced by nascent RNA as Pol II moves into the more stable PC (Sainsbury et al. 2013), Pol II PIP-seq, but not TFIIB, is expected to reveal the location of the PC. Comparison of TFIIB and Pol II PIP-seq should then separate PICs and PCs, respectively.

The transcription machinery also assembles at the TSS of many noncoding transcription units (ncRNA), which includes divergent TSSs that arise upstream of mRNA TSSs and are transcribed from the opposite strand (Core et al. 2008, 2014; Mayer et al. 2015; Scruggs et al. 2015). Transcriptional enhancers that reside far from annotated mRNA TSSs can also be transcribed, and it has been suggested that this transcription arises from specific points of initiation (Core et al. 2014). However, it is not known whether PICs and PCs exist in genes encoding ncRNA or whether they are similar to those at mRNA genes.

Here we use PIP-seq to separate human PICs from PCs on a genomic scale. We use this high-resolution data to assemble a more comprehensive view of how initiation complexes form and initiate transcription across a broad variety of promoter classes.

## Results

### PIP-seq validation

**Figure 1.**
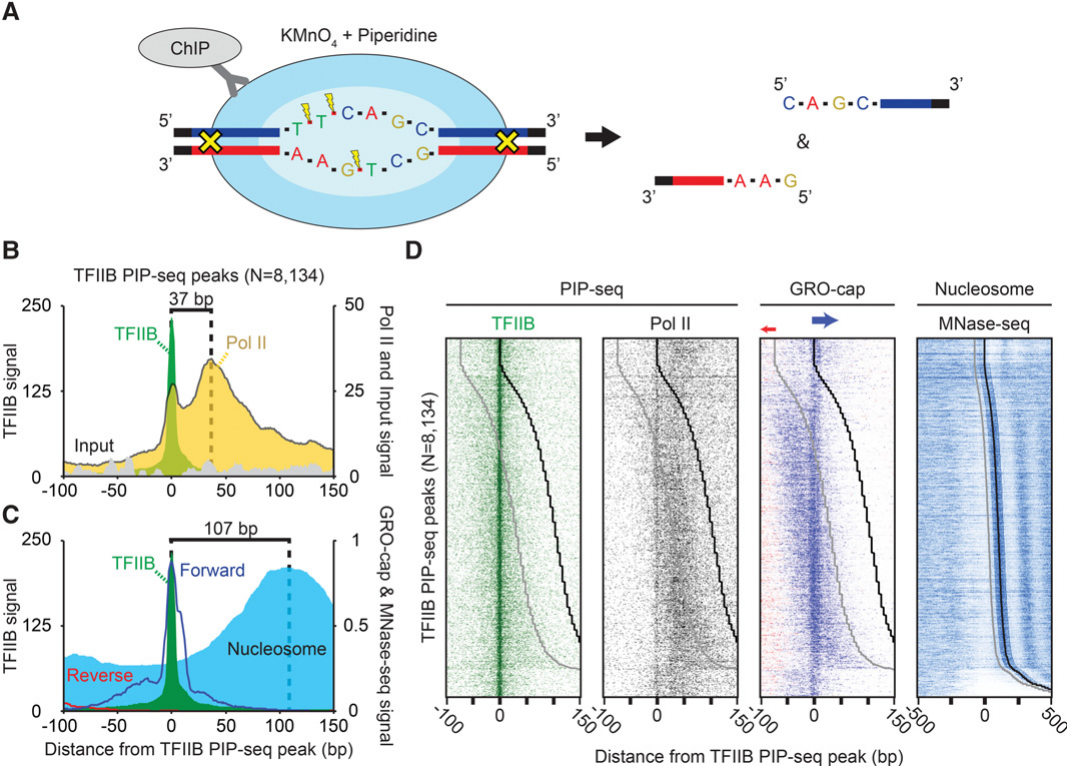
Positional separation of open preinitiation complexes (PICs) and paused complexes (PCs) associated with ncRNA and mRNA transcription. (*A*) Schematic of the PIP-seq assay. KMnO_4_ oxidizes single-stranded thymines, which are subsequently cleaved by piperidine. Coupled to formaldehyde-based crosslinking and immunoprecipitation, open DNA relative to a protein of interest is enriched. (*B*) Composite plots of PICs (*N* = 8134). TFIIB-bound open complexes were identified as enriched TFIIB PIP-seq peaks (see Methods) that also had a corresponding enrichment of TFIIB ChIP-exo peaks, as well as GRO-cap transcription. (*C*) Composite plots of PICs (*N* = 8134) overlaid with composite plots of GRO-cap (RNA) and MNase-seq tag 5′ ends (nucleosomes) that were shifted in the 3′ direction by 80 bp or approximately half the average fragment length (Lai et al. 2012; The ENCODE Project Consortium 2012; Core et al. 2014). (*D*) Heatmap of PICs (*N* = 8134), sorted by the distance between TFIIB PIP-seq peaks to downstream +1 nucleosomes. The black line represents the consensus +1 nucleosome dyad, and the gray line is 73 bp upstream of the dyad representing the upper edge of the nucleosome. The few dyads that align exactly on the TSS are likely artifacts resulting from parameter settings and thresholding and thus should be ignored.

Our objective was to separately detect, on a genomic scale, the open DNA associated with PICs and PCs in human K562 cells. K562 cells were selected due to an abundance of relevant data existing for this cell line. Open DNA can be “marked” in vivo because thymines are more readily oxidized by permanganate when they are single-stranded (Fig. 1A; Giardina et al. 1992). When combined with ChIP, these marked regions can be purified and linked to the immunoprecipitated protein (TFIIB or Pol II) (Supplemental Fig. S1; Li et al. 2013). Operationally, a sequencing adaptor is attached to free ends of the immunoprecipitated DNA. Piperidine is then used to cleave the DNA, just 3′ to the oxidized thymidine. Only one of the two strands are cleaved at each thymidine; the complementary strand remains intact unless it too has an oxidized thymidine nearby. The cleaved newly released 5′ ends are then deeply sequenced (Supplemental Table S1). The initial set of replicates for TFIIB and Pol II PIP-seq were used for peak calling and visualization in all plots due to their high signal-to-noise ratio, although all replicates correlated well compared to the input (Supplemental Fig. S2A). Moreover, described patterns were identical between biological replicates (Supplemental Fig. S2B).

Since the DNA backbone 3′ to a “T” nucleotide is preferentially cleaved in the PIP-seq assay, a concern is whether genomic DNA in putative PIC regions is intrinsically enriched with T's, thereby giving the false appearance of a PIC (false positive). We therefore analyzed annotated TSS regions (*N* = 26,163) (Pruitt et al. 2007) for intrinsic “T” nucleotide bias, particularly those having high levels of PIP-seq tags (Supplemental Fig. S3). For genes with multiple annotated transcription start sites, the most 5′ coordinate was selected. We found that such regions were not intrinsically biased toward “T” compared with TSS regions having low PIP-seq tags. Indeed, such regions are relatively depleted of “T” (and “A”) and instead are intrinsically enriched with “G” and “C,” which is in line with TSS regions known to reside within CpG islands (Deaton and Bird 2011). Thus, PIP-seq enrichment was not due to intrinsically T-rich TSS regions.

We also considered the corollary that intrinsically T-depleted regions would be refractory to PIP-seq (false negatives). In order to address this possibility, we called TFIIB-bound regions using two distinct criteria, TFIIB ChIP-exo peaks possessing enriched GRO-cap signal, which specifies active transcription start sites (RNA 5′ ends) (Core et al. 2014). We then examined the top 1000 peaks by TFIIB ChIP-exo signal and identified those sites that failed to meet a TFIIB PIP-seq threshold (Poisson *P* < 1 × 10^−4^). Less than 5% of the top 1000 ChIP-exo peaks failed to pass this threshold. Those 5% nevertheless had PIP-seq tags that exceeded the background (but missed our stringent threshold for being called a location) (Supplemental Fig. S3C). Thus, their status as “false negatives” is due primarily to high thresholding rather than being undetectable.

We next considered the possibility that parts of the PIP-seq assay workup (apart from permanganate treatment) caused a nucleotide bias. Steps with potential bias include formaldehyde crosslinking, chromatin fragmentation by sonication, nuclear extraction, ChIP, DNA ligation, PCR, and DNA sequencing. To this end, we compared the nucleotide composition at position −1 of tag 5′ ends in PIP-seq (where “T” is supposed to be enriched) to that of ChIP-exo (Supplemental Figs. S4A, S5A). In contrast to PIP-seq, tag 5′ ends from ChIP-exo correspond to exonuclease stops (due to blockage by a crosslinked protein) (Rhee and Pugh 2012a) and thus are not expected to have T-bias apart from what is intrinsic to DNA. Since both assays use essentially the same processing steps, with the exception of how tag 5′ ends are generated, they should correlate well in relative tag enrichment but differ in the exact position of the 5′ end of the sequence read and thus the identity of the −1 nucleotide.

As expected, both assays produced tag enrichment around TSSs (due to the ChIP aspect of the assay), including a bimodal distribution that has been attributed to divergent transcription (Supplemental Fig. S4B,C for TFIIB; Supplemental Fig. S5B,C for Pol II; Core et al. 2014; Scruggs et al. 2015). In the control comparison, TFIIB and Pol II ChIP-exo nucleotides at the −1 position of tag 5′ ends were depleted of A + T in the TSS region in accord with what is intrinsic to the DNA, rather than being biased toward “T” (Supplemental Fig. S4D for TFIIB; Supplemental Fig. S5D for Pol II). Thus, the processing steps that are in common with both ChIP-exo and PIP-seq were not creating a collective bias in nucleotide enrichment. In contrast, the TFIIB PIP-seq assay produced a strong −1 “T” enrichment in the TSS region, consistent with its permanganate reactivity. We therefore used only those tags having a “T” at −1 (relative to the tag 5′ end) for calling PIP-seq peaks and for plotting tag 5′ end distributions.

### Spatial separation of PICs and PCs

We selected for further study those TFIIB PIP-seq peaks that had a statistically enriched −1 “T” tag density compared to input DNA and that were within 100 bp of a TFIIB location defined by ChIP-exo (12,398 peaks). By using the same criteria, we also called TFIIB PIP-seq peaks that required enrichment of GRO-cap (Core et al. 2014) signal within 100 bp instead of TFIIB ChIP-exo (16,396 peaks). From the union of these sites, we identified with high confidence 8134 transcriptionally active TFIIB-bound open complexes. The positions of the 5′ ends of PIP-seq sequence tags for Pol II and the input were aligned to the center of the detected TFIIB PIP-seq peaks and orientated so that the maximal GRO-cap transcription signal was on the top strand (Fig. 1B, 5′-3′, left to right). In this analysis, we chose not to align by TSS since we found that such locations have their own positional uncertainty (e.g., see Supplemental Fig. S4C), which degraded the intrinsic pattern resolution.

Pol II PIP-seq 5′ ends produced two distinct peaks, the highest at the expected position of the PC. Remarkably, the distribution of the lower peak coincided exactly with a local peak of TFIIB PIP-seq peaks, denoting open DNA complexes. Thus, these overlapping peaks of TFIIB and Pol II mark the positions of PICs (Fig. 1B), which represent a separation of PICs and PCs across a genome at near-single base-pair resolution. Although PIC and pause separation was reported by Quinodoz et al. (Quinodoz et al. 2014; Waszak et al. 2015), we find the data are inconsistent with such locations (Supplemental Fig. S6). Our PIC location is also precisely where GRO-cap 5′ ends mapped (Fig. 1C). The distribution of Pol II PIP-seq tags was as tightly focused at TFIIB peaks as those of TFIIB (Fig. 1B), which provides further support that they are reporting on the same complex. Inasmuch as PIP-seq measures a steady-state population of open complexes, the location of the open DNA at the TSS represents a steady state. This is not necessarily where promoter melting initiates, which is thought to occur just upstream of the TSS (Guzman and Lis 1999).

The majority of Pol II PIP-seq tags were distributed in the pause region over a relatively broad range of ∼20–60 bp downstream from the TSS (Core et al. 2008; Adelman and Lis 2012). TFIIB PIP-seq tags were not enriched at Pol II pause sites. This demonstrates a lack of both direct and indirect crosslinking of TFIIB at the PC. It follows then that the detection of Pol II at the PIC site is not an artifact of the PC crosslinking indirectly via TFIIB. The lack of TFIIB at the PC also indicates that the PC lacks TFIIB in its active site. This is expected due to the presence of nascent RNA there (Kettenberger et al. 2004). The lack of TFIIB tags in the PC region provides further evidence that PIP-seq is not measuring some artefactual permanganate reactivity of the PC DNA (i.e., TFIIB serves as an additional negative control for Pol II). We therefore conclude that TFIIB and Pol II PIP-seq provide quantitatively robust and positionally accurate separation of the PIC and PC active sites across the human genome.

### Distinct specificities of focused versus dispersed initiation

PICs and PCs, defined by PIP-seq, typically resided within nucleosome-free regions and just upstream of an MNase-resistant +1 nucleosome (Fig. 1B,C). The position of the PC has been linked to the position of the +1 nucleosome, which has led to the conclusion that the PC and +1 nucleosome might influence each other's positioning at certain classes of genes (Mavrich et al. 2008; Gilchrist et al. 2010; Li and Gilmour 2013; Teves et al. 2014; Weber et al. 2014). With the higher resolution afforded by PIP-seq, we reexamined the relationship between Pol II at the 5′ ends of genes and the neighboring +1 nucleosome. In doing so, GRO-cap (transcription initiation) and ENCODE K562 MNase-seq (assumed nucleosome occupancy) tags were aligned relative to the TFIIB PIP-seq peak (The ENCODE Project Consortium 2012; Core et al. 2014) at individual mRNA genes and sorted by the distance between TFIIB and the +1 nucleosome (Fig. 1D). The peak of GRO-cap 5′-end signal on the forward strand aligned precisely with the peak of TFIIB-bound open DNA (PIC), regardless of nucleosome positioning. Similar but weaker conclusions could be drawn when aligned by GRO-cap and CAGE data (Supplemental Fig. S6; The ENCODE Project Consortium 2012; Core et al. 2014). Thus, the predominant PIC position and its corresponding PC do not appear to be tightly linked to the +1 nucleosome position.

We observed GRO-cap 5′ ends in a disperse pattern around the primary PIC location (Fig. 1D), thereby indicating that dispersed and focused transcription initiation are largely colocalizing within promoters containing a stable PIC. Approximately 93% of detected PICs possessed significant (Poisson *P* < 1 × 10^−4^) GRO-cap tags in both a focused (±10 bp) window around the PIC, as well as a wider dispersed window (40-bp window immediately flanking the focused region). This differs from the notion that focused and dispersed transcription initiation represents distinct gene classes (Carninci et al. 2006; Kawaji et al. 2006; Juven-Gershon et al. 2008; Juven-Gershon and Kadonaga 2010), which may be reconciled by differences in assay sensitivity and/or data thresholding.

Surprisingly, and in contrast to the primary TSS, dispersed initiation tracked with the position of +1 nucleosomes (Fig. 1D). Moreover, dispersed initiation was not equivalently mirrored by TFIIB or Pol II PIP-seq data, which largely reflected the primary PIC and PC events, respectively. Thus, dispersed PICs and PCs may not be as stable as focused PICs and PCs.

We next sought additional positional cues of focused and dispersed initiation by searching for DNA motifs present in the JASPAR database. These motifs were enriched immediately upstream of the focused PIC, in line with a similar report (Supplemental Fig. S7A; Scruggs et al. 2015). However, we also note that the relative motif enrichment tracked poorly with the dispersed initiation (Supplemental Fig. S7B). Taken together, these results suggest that dispersed transcription initiation is tied primarily to +1 nucleosome positions and less to transcription factor positions. These data support a model previously described in zebrafish (Haberle et al. 2014), where relatively transient and dispersed PICs may be initiating at a large number of positions in a transcriptionally permissive, accessible environment that is bounded downstream by the position of the +1 nucleosome. The source of an upstream boundary is unclear. These results of comparing motif enrichment, dispersed initiation, and nucleosome position do not establish causality or direct interactions. The notion that transcription can initiate in multiple potential areas in a core promoter (Kawaji et al. 2006; Core et al. 2014) but still have a dominant start site (Scruggs et al. 2015) is supported here but further suggests that at least some aspects of focused versus dispersed initiation occur by distinct mechanisms.

### Pol II directionality independent of RNA measurements

**Figure 2.**
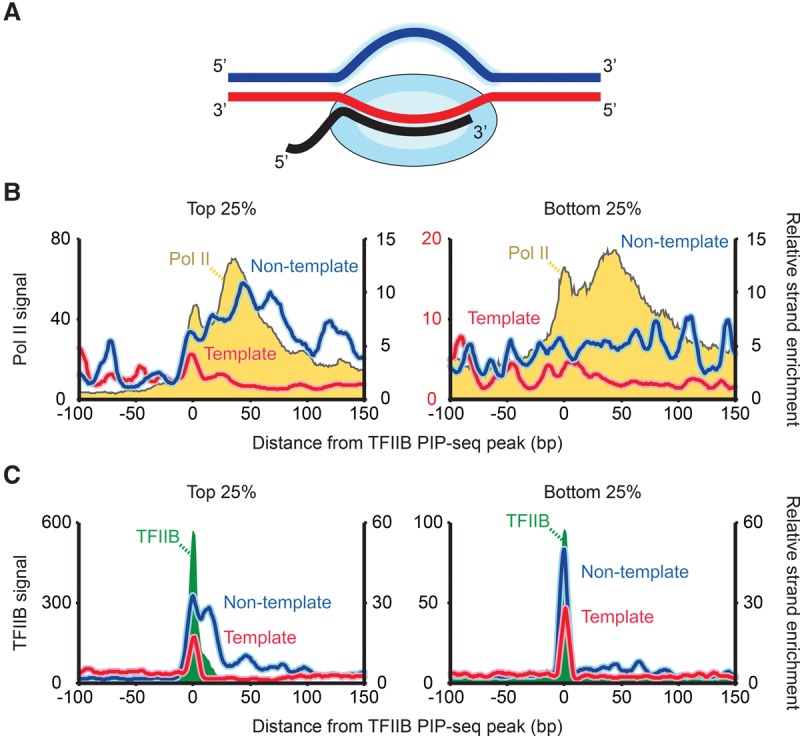
Pol II directionality measured by PIP-seq. (*A*) Cartoon depicting variable solvent accessibility at the Pol II active site driven by the presence of the transcribing complex and RNA. (*B*) Composite plot of Pol II PIP-seq tags separated by the top and bottom 25% of TFIIB PIP-seq occupancy (yellow) overlaid by strand-separated Pol II PIP-seq tags (blue and red). Separate strands are normalized by corresponding strand-separated −1 “A” tags to remove ChIP signal and leave only specific strand enrichment. The *y*-axes are scaled differently between the top and bottom 25% in order to better visualize the relative enrichment patterns. (*C*) Same as panel *B* but for TFIIB PIP-seq (green).

One of the challenges in genome-wide studies of early transcription elongation is defining the location of Pol II prior to it synthesizing sufficient RNA to uniquely map it in a genome. Because PIP-seq is a DNA-based assay, it does not require long, relatively stable RNA transcripts to identify elongating polymerase. Furthermore, since permanganate oxidizes single-strand thymines, PIP-seq should produce cleavage events preferentially on the nontemplate DNA. Therefore, if RNA is base-paired to its template DNA within the Pol II active site, then “T” nucleotides within this RNA/DNA hybrid are expected to be less reactive to permanganate than on the nontemplate (sense) strand (Fig. 2A). Moreover, since this is related to transcription, the strand bias of “T” reactivity should be stronger at highly transcribed genes.

To test for strand bias, we generated composite plots of Pol II PIP-seq within the top and bottom quartile of TFIIB PIP-seq signal (Fig. 2B). To remove enrichments caused by ChIP and nonspecific piperidine cleavage, we normalized the permanganate-sensitive “−1 T” PIP-seq tag density to those of “−1 A,” which occurs at the same frequency as “T” but is insensitive to permanganate (Supplemental Figs. S4, S5). We confirmed the validity of this normalization by performing an equivalent PIP-seq data normalization of “−1 G” with “−1 C,” both of which reflect ChIP signals (Supplemental Fig. S8A), and also by performing an equivalent ChIP-exo normalization (Supplemental Fig. S8B). In all cases, the ChIP signal became fully normalized, as evidenced by the ratio around the TSS becoming essentially 1.0 (i.e., flat-lined traces at *y* = 1).

In accord with our strand-bias hypothesis, we observed substantially more Pol II PIP-seq tags on the nontemplate strand, particularly at highly transcribed genes (Fig. 2B, left panel, blue vs. red trace for top 25% of TFIIB PIP-seq signal). This bias of Pol II predominated immediately downstream from the TFIIB PIP-seq PIC location, which is where nascent RNA pairs with the template strand. The diminished bias at the PIC likely reflects the relative absence of RNA in the Pol II PIC active site.

A similar diminished bias was observed for TFIIB PIP-seq tags at the PIC site (Fig. 2C). However, a nontemplate strand bias was observed further downstream by ∼20 bp at highly transcribed genes (Fig. 2C, left panel). Beyond this distance, TFIIB was not particularly enriched, and so any residual bias may have little meaning. We speculate that the bias out to ∼20 bp may be caused by RNA–DNA hybridization protection within Pol II, perhaps through abortive initiation occurring while TFIIB is still within a crosslinkable distance to Pol II (Cabart et al. 2011; Sainsbury et al. 2013). That positional relationship is assumed to be lost further downstream within the PC.

The bias in “T” reactivity toward the nontemplate strand suggests that it might be used to define the direction of transcription, which would be particularly useful in its early stages where RNA is too short to uniquely map to the genome. To test this idea, we attempted to predict the direction of transcription for our 8134 TFIIB-defined PIC regions using the relative “−1 T” tag enrichment of Pol II PIP-seq data on the sense strand in the two candidate directions (i.e., the two 3′ directions relative to the PIC). In order to prevent the strand orientation prediction from being biased by divergent transcription, we compared the relative strand enrichment in a window smaller than the minimum distance observed for divergent transcription (<100 bp) (Core et al. 2014). We successfully predicted the direction of transcription 76% of the time, using GRO-cap as the gold standard for directionality. That number climbed to 85% for the top 25% TFIIB-bound initiation complexes. The number expected by chance is 50%, which is what we observed (53%) when using “−1 A” tags as a negative control. Beyond PIP-seq measurement error, the upper bounds of our estimates are necessarily limited by several external factors, including GRO-cap measurement error and the direction of transcription being a binary assessment rather than a scaled differential that occurs in a population of molecules.

### PIC and PC open complex organization is similar at ncRNA and mRNA promoter regions

While previous studies have shown the presence of the transcription machinery where ncRNA is produced (Core et al. 2014; Mayer et al. 2015; Nojima et al. 2015), it has not yet been investigated to what extent ncRNA PICs versus PCs exist and their level of similarity to those at mRNA genes. We therefore investigated whether the PIC and PC were differentially represented at the promoter regions of ncRNA compared with mRNA. For this purpose, we focused on distal ncRNA that had its TSS being >1 kb from an annotated coding mRNA TSS. The resulting 2660 distal sites of transcription initiation were enriched for ncRNA production sites in enhancers.

Confirming their enrichment in enhancers regions, these ncRNA locations are predicted to be enhancers (53%) by ChromHMM (Supplemental Fig. S9A; Ernst et al. 2011) and are enriched with enhancer-associated EP300 (Supplemental Fig. S9B; The ENCODE Project Consortium 2012). Moreover, while being far from annotated TSSs, their nearest annotated genes tended to be involved in hematopoietic functions (Supplemental Fig. S9C; McLean et al. 2010). This is consistent with many of them being enhancers of genes that specify the myeloid origin of the K562 cell line used in these experiments. In contrast, the TFIIB-bound mRNA promoters were enriched for housekeeping processes (transcription and translation). Taken together, these findings confirm that this collection of putative distal ncRNA PICs largely reside in enhancers and likely represent eRNAs. The ability of PIP-seq to identify transient transcriptional events such as eRNA production emphasizes its high sensitivity and its role as a potential complement to RNA-based assays, when uniquely mapping transcripts <20 bp in length to the genome (Scruggs et al. 2015) or where RNA is highly unstable.

**Figure 3.**
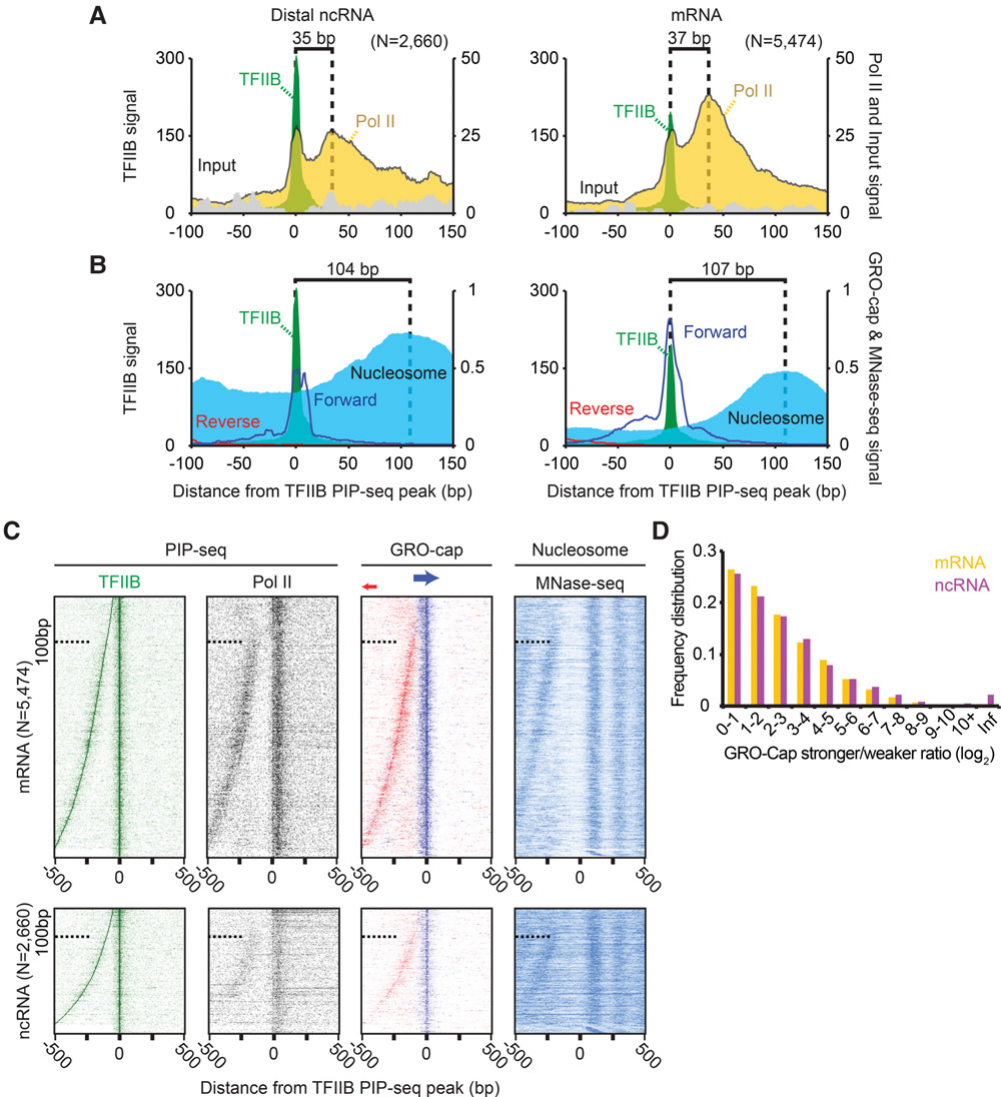
Equivalence of ncRNA and mRNA initiation complexes organization. (*A*) Composite plot of TFIIB, Pol II, and input PIP-seq at TFIIB PIP-seq peaks separated by ncRNA and mRNA proximity. Locations were considered ncRNA-associated (*left* panel; *N* = 2660) if located >1 kb from an annotated mRNA TSS and considered mRNA-associated (*right* panel; *N* = 5474) if <1 kb away. Dashed lines represent the distance between the TFIIB and Pol II PIP-seq local maxima. (*B*) Same as panel *A*, but displaying strand-separated GRO-cap RNA (blue and red lines) and nucleosomes. Dashed lines represent the distance between the TFIIB PIP-seq and MNase-seq local maxima. (*C*) Heatmap of TFIIB and Pol II PIP-seq, GRO-cap RNA, and nucleosomes sorted by the distance between the TFIIB PIP-seq peak and the closest TFIIB PIP-seq local maxima located between 50 and 500 bp upstream. (*D*) The log_2_ ratio of GRO-cap RNA on opposing strands (stronger/weaker) was binned, separated into mRNA and ncRNA classes, and plotted as a frequency distribution. Initiation complexes were assigned infinity (Inf) if there was no detected GRO-cap signal on one of the strands.

PICs and PCs were detected at both mRNA and distal ncRNA transcription units, with the pause distance being similar in both classes (Fig. 3A). However, PICs were proportionally more abundant relative to PCs at ncRNA than at mRNA. We interpret this as a result of either relatively greater stability of the ncRNA PIC and/or lower stability/formation of its PC. The known instability of ncRNA is consistent with the latter interpretation (Preker et al. 2008), but this assumes that RNA and PC instabilities are linked. GRO-cap RNA 5′ ends also aligned to the ncRNA TFIIB PIP-seq peaks, albeit with lower precision and abundance compared with mRNA genes (Fig. 3B). ncRNA transcription units also had a canonically positioned +1 nucleosome. Together these findings suggest that ncRNA PICs are organized similarly to mRNA PICs, although once initiated, their PC counterparts may be relatively unstable.

We identified TFIIB-bound open complexes just upstream of our detected mRNA and ncRNA PICs that matched the divergent, or bidirectional, transcription that has been previously characterized with RNA-based assays (Fig. 3C; Core et al. 2014; Duttke et al. 2015a; Mayer et al. 2015). These upstream open-complexes were also transcribed and linked to the positioning of a nucleosome downstream from its transcription as in the other TFIIB-bound open complexes. We further found that such divergent PICs could be no closer than ∼100 bp from each other. Although TFIIB PIP-seq signal existed beneath this distance range, it was not validated by Pol II PIP-seq and GRO-cap, indicating that they were not bona fide PICs (see dashed line in each top of each panel in Fig. 3C), which is in agreement with previous results showing an average 110-bp distance between GRO-cap initiation (Core et al. 2014).

Transcription at mRNA genes is, on average, more frequent in the mRNA direction (Duttke et al. 2015a) but is thought to be roughly equivalent in both directions at the enhancers (Andersson 2015). We calculated the stronger/weaker log_2_ ratio of GRO-cap signal in the two directions and found the genomic variance in the divergence ratios to be essentially the same regardless of whether an mRNA was being synthesized (Fig. 3D). Thus, while the degree of bidirectionality can vary (Andersson et al. 2015; Duttke et al. 2015b), we find that this variance is equivalent at enhancers and mRNA promoters containing stable PICs.

### A blend of DNA sequence and structure define PIC locations

The accuracy and resolution of TFIIB PIP-seq in identifying PIC locations allowed us to examine the underlying DNA sequence features of PICs, which have been rather elusive at most promoters. Not surprisingly, MEME-ChIP analysis identified the known INR-element (YYANWYY) (Smale and Baltimore 1989) as the most overrepresented sequence. However, even using an extremely lenient motif *P*-value threshold of 1 × 10^−2^, which is two orders of magnitude less than the MEME suite software default, and a search space of 20 bp, FIMO found the INR motif at only ∼20% of the TFIIB-bound PICs, at both ncRNA and mRNA promoter regions. Thus, either the underlying PIC DNA generally lacks a sequence signature or the MEME model for motif searching was inadequate. To consider the latter possibility, we took a different approach by using a normalized log-likelihood ratio (Stormo 2000) to quantify all 7mer sequences within ±50 bp of a PIC according to how closely they resemble an INR consensus. As a negative control, the sequence in the same search space was scrambled and run in parallel.

**Figure 4.**
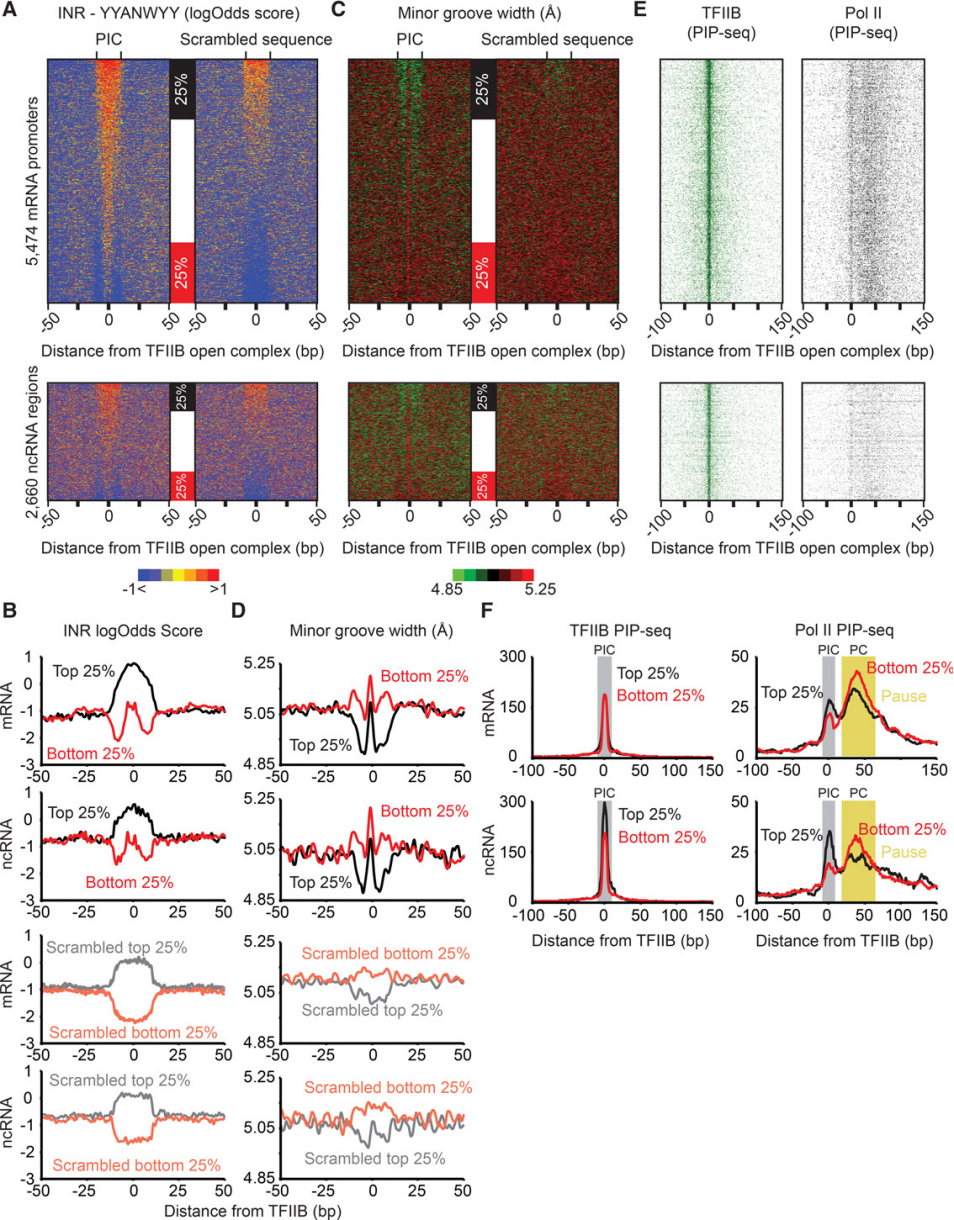
Genomic DNA architecture of PICs. (*A*) Genomic sequences (100 bp in each row) surrounding TFIIB PIP-seq peaks (*N* = 8134) or their scrambled 100-bp counterparts were scanned with the YYANWYY (IUPAC nomenclature) (Smale and Baltimore 1989) consensus in a sliding window to calculate log-likelihood ratios (Stormo 2000). Rows for the PIC and scrambled sequence were then sorted independently of each other based on the average score in a 20-bp window that was centered on each TFIIB peak. (*B*) The top and bottom 25% of rows from panel *A* were used to generate composite plots. (*C*) Minor groove widths were calculated (Zhou et al. 2013) for the sequences defined in panel *A* and sorted based on panel *A*. (*D*) The top and bottom 25% of rows from panel *C* were used to generate composite plots. (*E*) TFIIB and Pol II PIP-seq tags were aligned and sorted based on panel *A*. (*F*) The top and bottom 25% of rows from panel *E* were used to generate composite plots. The initiation and pause regions are highlighted in gray and yellow, respectively.

Those 7mer sequences that were the most similar to an INR within the local search space showed particular enrichment within ±3 bp of a TFIIB PIP-seq-defined PIC (Fig. 4A, sorted by log-odds score and quantified in Fig. 4B). The same was true at both mRNA and ncRNA open complexes and was not observed for the scrambled control sequences.

Surprisingly, the centering of the locally best 7mer precisely on TFIIB PIP-seq peaks was quite evident, even where the absolute INR-like scores were no different than the random average (bottom 25% in Fig. 4A,B, as demarcated by red boxes). In these cases, the flanks were enriched with very low scoring 7mers, well below the genomic average, and thus may have “anti-INR” character. No such centering pattern was observed with the scrambled control sequences. Thus, in a large proportion of cases, the open complexes were residing at the best INR-like sequence in the local vicinity, despite them being essentially no different from a random sequence on a genomic scale. Such elements would therefore have only local context specificity since the surrounding sequence may be more refractory to maintaining the open strand-separated state. If true, INRs (focused initiation) might utilize globally nonspecific sequences embedded in an “anti-INR” environment, thereby making them locally specific.

Those INR-like 7mers that scored the highest on an absolute scale (Fig. 4A, top 25% of the upper and lower panels; Fig. 4B, black traces) had a relatively broad enrichment compared with the bottom 25%. Since Pol II interacts with the minor groove of DNA within the open complex (Barnes et al. 2015), we investigated whether changes in minor groove width might provide additional local specificity. For this, we used a computational DNA shape predictor (Zhou et al. 2013). We observed that PIC regions having the highest and broadest stretch of log-likelihood INR similarity based on DNA sequence (Fig. 4A, top 25% of the upper and lower panels; Fig. 4B, black traces), and thus would seem to have less local discrimination, were nonetheless enriched with a narrowly focused short stretch of relatively wide minor groove (Fig. 4C, top 25% of the upper and lower panels; Fig. 4D, black traces). This enrichment was concentrated within a few base pairs of PIC locations (TFIIB PIP-seq peaks) and flanked by local regions having a narrower minor groove.

One characteristic of these high-scoring INR-like regions was not so much a wide minor groove on an absolute scale (they were about average), but instead, their flanks were predicted to have narrower minor grooves (Fig. 4D). This pattern was analogous to what was observed in regions having essentially random INR-like scores (Fig. 4A) but flanked by anti-INR sequences.

Taken together, these results suggest that PICs largely reside on “average nonspecific” DNA that is flanked by local DNA having properties that comparatively resist supporting an open complex. This resistance may include an avoidance of INR-like sequences and having a narrower minor groove. Importantly, this is a local property that is not likely to provide global recruitment specificity, but rather provides local specificity. Nevertheless, a small but substantial fraction of open complexes do reside at consensus INR elements. We envision a genome-wide continuum whereby some balance of core INR-like sequence and minor groove shape are flanked by opposing sequences so as to stand out and provide local specificity. Since we did not observe differences in PIC occupancy at strong versus weak INRs (Fig. 4E), we surmise that the entire continuum accommodates PIC formation. However, the PIC/PC ratio was higher where PICs were embedded in relatively strong INR-like sequences (Fig. 4F, right panels). Therefore, INR-like sequences might regulate PIC-to-PC conversion, particularly if they influence transcription initiation efficiency.

## Discussion

Pervasive transcription has diverse functions throughout the genome, and is typically studied through RNA-based assays (Core et al. 2014; Mayer et al. 2015; Nojima et al. 2015; Scruggs et al. 2015). These assays, while sensitive and of high resolution, are limited in requiring ∼20 bp or more of transcribed RNA for unique mappability to the mammalian genome. PIP-seq, in contrast, involves DNA fragmentation to >100 bp, and so the generated fragment sizes do not suffer from mappability concerns. Moreover, the 5′ ends of PIP-seq reads identify open complexes at single-nucleotide resolution. We find that PIP-seq is capable of identifying open PICs genome-wide at a resolution that exceeds the high-resolution DNA-based ChIP-exo assay. Although PIP-seq relies on the presence of a single-stranded thymine in an open complex for detection, the overall quantity of T's did not affect the signal at detected complexes. While RNA-based assays are well suited for assigning directionality of a transcribing complex, in situations where insufficient RNA is available, PIP-seq takes advantage of the differential permanganate reactivity of the template (being hybridized to RNA) and transcribed strand so as to define the orientation of Pol II on the DNA.

Our analyses of TFIIB and Pol II PIP-seq data reveal the capability of PIP-seq to spatially separate PICs from PCs, further supporting the assay's high sensitivity. At mRNA genes, steady-state levels of PCs far exceed PIC levels, which supports the notion that PICs are rapidly converted to PCs. This contrasts with ncRNA promoters where PCs are relatively less abundant, perhaps owing to greater ncRNA/PC turnover. Nevertheless, we find that ncRNA, whether arising from enhancer regions or from divergent transcription upstream of mRNA promoters, is associated with PICs and PCs in a manner that is qualitatively no different than at mRNA promoters. This includes unequal levels of transcription in the two directions, which contrasts with views of enhancers that assume directional equality when performing enhancer averaging (Andersson et al. 2014). At mRNA genes, transcription is often more active in the sense direction compared with upstream in the divergent direction. This ratio of transcription in the two directions, however, varies considerably from gene to gene (Duttke et al. 2015a). The same is true at enhancers, where transcription in one direction is typically more active than in the divergent direction.

**Figure 5.**
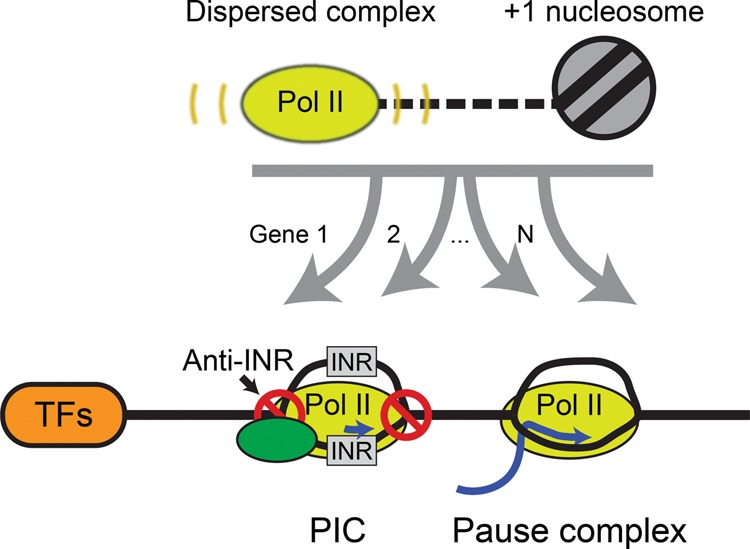
Alternative models of transcription initiation. Cartoon model displaying the proposed regulatory factors of diffuse and focused transcription at TFIIB-bound initiation complexes. After initial recruitment of the initiation complex to a region, initiation may occur at the optimal TSS (focused initiation). Dispersed nucleosome-linked initiation may result from the presence of a transcriptionally permissive region with a downstream nucleosome serving as a boundary element. Alternatively, disperse transcription may operate in a local region resulting in a positioned downstream nucleosome.

Upon examination of GRO-cap data in relation to our high-resolution PICs, we identified focused and dispersed transcription initiation events (Fig. 5; Juven-Gershon et al. 2008; Juven-Gershon and Kadonaga 2010). In contrast to previous reports that these two modes of transcription occurred at different promoters, we find them co-occurring within the same promoter (Carninci et al. 2006; Lenhard et al. 2012). However, they are not likely to be simultaneously on the same promoter DNA molecule, due to steric occlusion.

Intriguingly, while dispersed transcription initiation sites appear to be linked to positions of the +1 nucleosomes, this is not the case for focused initiation sites. We suspect that distinct mechanisms give rise to these two classes, although they are not necessarily independent events. When examining the underlying DNA sequence at focused initiation complexes, the TFIIB-bound PICs were enriched for an INR-like sequence relative to the local sequence. This ranged from canonical INRs to random sequences that were flanked by anti-INR sequences. Additionally, regions that possessed a broader set of INR-like DNA were indeed further discriminated by a locally wide minor groove flanked by DNA having a relatively narrow minor groove. While we did identify TATA-elements upstream of PICs at the expected location (∼30 bp), we did not find them enriched beyond previously described levels (Carninci et al. 2006). We also identified enrichment for known vertebrate transcription factor DNA motifs having positions immediately upstream of and positionally linked to focused PICs, thereby implicating them in forming PIC (and PC) positions, which contrasts to an apparent lack of linkage to dispersed initiation.

The combination of dispersed nucleosome-linked transcription initiation surrounding a focused site of transcription at the locally best core INR-like sequence (Fig. 5) offers two general mechanisms of PIC assembly. Both mechanisms are assumed to involve activators recruiting PIC components to generally accessible promoter regions as the primary determinant of activation, although that aspect is not addressed here. Beyond recruitment, in the first mechanism PIC components recognize INR-like sequences and minor groove architecture to set the location of relatively stable open DNA and thus PIC positioning. This creates a focused transcription initiation site and is defined by DNA sequence and shape. The focused site is not necessarily where DNA melting initiates but rather is where it is sufficiently long lived to detect and direct transcription initiation. This focused initiation is coupled to a PC over a variable distance (20–60 bp) downstream, which is mostly upstream of an adjacent +1 nucleosome. In accordance with a model proposed in *Drosophila* (Kwak et al. 2013), we observed that the +1 nucleosome is not positionally linked to the predominant PC density and thus is not likely to be the primary barrier that defines the pause site for PCs that arise from focused initiation, nor a means to position the +1 nucleosome.

The second initiation mechanism derives from the observation that dispersed GRO-cap transcription initiation sites were positionally linked to +1 nucleosome positions and thus raises the question of causality. From one perspective, +1 nucleosome positioning might establish PIC positioning, akin to a yeast model (Rhee et al. 2014). Thus, dispersed +1 positioning creates dispersed PICs, possibly through direct engagement or by establishing accessibility barriers. Studies in yeast showing chromatin-mediated regulation of divergent transcription further support the role of nucleosome positioning in regulating levels of diffuse transcription (Marquardt et al. 2014). From a reciprocal perspective, dispersed initiation events might occur first, thereby leading to +1 nucleosome positioning, akin to what is suggested in *Drosophila* (Gilchrist et al. 2010). If transcription initiation drives nucleosome positioning, such nucleosomes would need to remain stable in the relatively long time intervals between initiation events. This seems inconsistent with promoter-proximal nucleosomes being quite dynamic (Dion et al. 2007; Rhee et al. 2014).

While we assume that dispersed PICs actually form, we were unable to detect them or a downstream PC distinct from the one linked to the focused PIC using PIP-seq. We do not think this is entirely an issue of technical sensitivity since dispersed and focused initiation as measured by GRO-cap occurred with similar frequency, and so trends observed with GRO-cap should have been evident in the PIP-seq data. Instead we are led to surmise that PICs and PCs from dispersed initiation either do not form at all or are very short-lived compared with focused PICs and their downstream PCs. Another possibility is that PC formation resulting from dispersed initiation occurs at the same location as the PC arising from focused initiation.

Our analysis is not inconsistent with prior work in *Drosophila*, where the +1 nucleosome was deemed to be a barrier to Pol II transit (Teves et al. 2014; Weber et al. 2014). That study was based on the positional enrichment of short Pol II–associated nascent RNA 3′ ends with +1 nucleosome positions that were also reported to be distinct from pausing. These “stall” sites generally occurred downstream from where Pol II pauses. Accordingly, Pol II likely encounters a second nucleosomal-based barrier after the primary pausing event, which involves nonnucleosomal pausing factors. This second barrier appears small relative to that encountered upon pausing.

## Methods

### Generation and sequencing of PIP-seq and ChIP-exo libraries

#### PIP-seq

PIP-seq was performed as previously described (Li et al. 2013) with chromatin from 50 million K562 cells (ATCC) grown in standard conditions (DMEM) and crosslinked with 1% formaldehyde. ChIP was performed in a volume of 750 µL after lysis and sonication, using 9 µg of TFIIB antibody (Santa Cruz, sc-225) and 3 µg of Pol II antibody (Santa Cruz, sc-899), respectively, conjugated to Protein G MagSepharose beads (GE Healthcare). Libraries were amplified with 16 cycles of PCR before size selection by gel excision.

#### ChIP-exo

ChIP-exo was performed as previously described (Rhee and Pugh 2012a) with chromatin from 20 million K562 cells (ATCC) grown in standard conditions and then treated with 1% formaldehyde prior to lysis and sonication. Three micrograms of Pol II antibody (Santa Cruz, sc-899) and 10 µg of TFIIB antibody (Santa Cruz, sc-225) were conjugated to Protein G MagSepharose beads (GE Healthcare) and used for ChIP. ChIP-exo libraries were amplified using 18 cycles of PCR followed by size selection by gel excision.

#### DNA sequencing

PIP-seq libraries were sequenced on a HiSeq 2000 producing 40-bp single-end reads (tags). ChIP-exo libraries were sequenced on an Illumina NextSeq 500, producing (2 × 40 bp) paired-end reads. All alignments to hg19 were performed with BWA using default parameters (Li 2013). Alignment to hg38 is not expected to significantly alter conclusions because findings were not dependent on alternative contig alignment. All PIP-seq aligned reads were then filtered such that all subsequent analysis and visualization was performed on reads that possessed a −1 5′ “T” unless otherwise stated.

### TFIIB-bound PIC calling

The genetrack peak-caller was run on TFIIB PIP-seq reads using s5 e20 parameters and a tag cutoff equivalent to *P* < 1 × 10^−5^ Poisson probability (Albert et al. 2008). Initial peaks were then filtered to remove known blacklist regions and peaks containing a significant (*P* < 1 × 10^−6^) amount of input PIP-seq tags (The ENCODE Project Consortium 2012). Putative TFIIB-bound peaks were further filtered by TFIIB ChIP-exo tags using a tag cutoff equivalent to a *P* < 1 × 10^−4^ Poisson probability in a 100-bp window around the peak. TFIIB PIP-seq peaks were then filtered to retain those that were associated with transcriptional activity, using GRO-cap tag counts in a 100-bp window around each TFIIB PIP-seq peak with a Poisson cutoff of *P* < 1 × 10^−5^. The strand of each peak was assigned based on the higher GRO-cap strand signal. PICs were next filtered with a 100-bp exclusion zone relative to each other, resulting in 8134 total peaks. For mRNA and ncRNA comparisons, peaks were split into two separate groups based on their proximity (within ±1 kb) to RefSeq TSSs using BEDTools (Pruitt et al. 2007; Quinlan and Hall 2010). This resulted in 5494 mRNA peaks and 2640 ncRNA peaks.

### TFIIB ChIP-exo peak calling

TFIIB ChIP-exo strand-specific peaks were called using the genetrack peak-caller with the s5 e20 parameters on the separate forward and reverse strands (Albert et al. 2008). Strand separate peaks were then paired and merged into a single peak under the requirement they be 0–80 bp 3′ from each other on separate strands. Strand separate peaks that did not possess a paired mate were excluded from further analysis. TFIIB ChIP-exo peak pairs were then filtered using GRO-cap tag counts in a 100-bp window around each TFIIB ChIP-exo peak pair with a Poisson cutoff of *P* < 1 × 10^−5^. Peaks were then split by a TFIIB PIP-seq tag cutoff equivalent to *P* < 1 × 10^−5^ Poisson probability into TFIIB ChIP-exo peaks passing PIP-seq threshold and those that did not. One thousand random coordinates were generated using the *bedtools random* command on the hg19 genome (Quinlan and Hall 2010). Both sets of peaks were filtered to remove known blacklist regions (The ENCODE Project Consortium 2012).

### Nucleosome calls

MNase-seq data from K562 cells were downloaded from the ENCODE Project Consortium (ENCSR000CXQ), and all biological replicates were merged (Kundaje et al. 2012). Aligned reads were shifted 80 bp from the 5′-to-3′ direction and piled up relative to all called PICs. Piled tags were subsequently smoothed using an 80-bp sliding window. The +1 nucleosome was then defined as the first local maxima detected downstream from the PIC.

### Detection of enriched motifs

Position weight matrices (PWMs) of the JASPAR 2016 vertebrate motifs were downloaded from the JASPAR database (Mathelier et al. 2016). Sequences were extracted in a 2-kb window around called PICs, and a control data set was generated by scrambling those sequences. PWMs were scanned across both data sets, with a *P* < 1 × 10^−4^, using FIMO at default parameters (Grant et al. 2011). Motif hits were then aligned relative to PICs.

### PIP-seq transcriptional orientation assignment

The 8134 called PIC regions were assigned a random strand orientation. Transcription directionality was then predicted by comparing the sum of −1 “T” reads from the downstream (20 to 100 bp) forward strand of the PIC to the sum of the reads upstream (−20 to −100 bp) reverse strand of the PIC. If the higher sum existed downstream, the random orientation was maintained; otherwise, the orientation was switched. The identical analysis was performed using the −1 “A” reads as a negative control. The GRO-cap predicted orientation was used as the gold standard for comparison.

### Detection of divergent PIC formation

Divergent PICs were identified by searching for the local TFIIB PIP-seq tag maxima 50–500 bp upstream of the called TFIIB PIP-seq peak. The log_2_ ratio of divergent transcription was determined by calculating the absolute value of the log_2_ ratio between the maximum GRO-cap tag peak of the forward and reverse strand in a ±500-bp window relative to the TFIIB called PIC.

### INR-element detection

Sequences in 20-bp windows around the TFIIB PIP-seq peaks were extracted using BEDTools (Quinlan and Hall 2010) and scanned for overrepresented motifs using MEME-ChIP (Machanick and Bailey 2011) with *P* < 1 × 10^−4^ and default parameters. The control data set was generated by randomly scrambling the DNA sequence of the PIC regions in order to remove nucleotide position information while retaining the local nucleotide content. Log-likelihood ratio (Stormo 2000) scoring was performed on 100-bp window of DNA sequences centered on the TFIIB PIP-seq peaks, comparing similarity to the INR consensus motif (YYANWYY) (Smale and Baltimore 1989) using a custom Perl script. Importantly, the local nucleotide background was calculated for each row independently. The log-likelihood score for each base pair is the higher of the score for the forward- and reverse-complement strand at the same position. Heatmaps were then sorted by the average INR log-likelihood ratio in a peak-centered 20-bp window.

### DNA-shape analysis

Minor groove width prediction was performed on a 100-bp window centered on TFIIB PIP-seq peaks for each peak using data from the DNAShape webserver (Zhou et al. 2013).

## Data access

All sequencing files and peak files from this study have been submitted to the NCBI Gene Expression Omnibus (GEO; http://www.ncbi.nlm.nih.gov/geo) under accession number GSE76955. Custom code used in analysis with sample inputs is available in the Supplemental Material (Supplemental Code).

## Competing interest statement

B.F.P. has a financial interest in Peconic, LLC, which utilizes the ChIP-exo technology implemented in this study and could potentially benefit from the outcomes of this research.

## Supplementary Material

Supplemental Material
