# Supplementary material for: Genome-wide uniformity of human ‘open’ pre-initiation complexes
Source: Genome Res. 2017 Jan;27(1):15–26. doi: 10.1101/gr.210955.116 (PMC5204339; doi:10.1101/gr.210955.116)
Supplement: Supplemental Material [file supp_27_1_15__index.html]

Genome-wide uniformity of human ‘open’ pre-initiation complexes — Genome-wide uniformity of human ‘open’ pre-initiation complexes — Supplemental Material 

# Genome-wide uniformity of human ‘open’ pre-initiation complexes

## Supplemental Material

- Supplemental\_Code.tar.gz
- Supplemental\_Fig\_S1.pdf
- Supplemental\_Fig\_S2.pdf
- Supplemental\_Fig\_S3.pdf
- Supplemental\_Fig\_S4.pdf
- Supplemental\_Fig\_S5.pdf
- Supplemental\_Fig\_S6.pdf
- Supplemental\_Fig\_S7.pdf
- Supplemental\_Fig\_S8.pdf
- Supplemental\_Fig\_S9.pdf
- Supplemental\_Fig\_S10.pdf
- Supplemental\_Table\_S1.pdf
