# Supplementary material for: Genome-wide uniformity of human ‘open’ pre-initiation complexes
Source: Genome Res. 2017 Jan;27(1):15–26. doi: 10.1101/gr.210955.116 (PMC5204339; doi:10.1101/gr.210955.116)
Supplement: Supplemental Material [file supp_gr.210955.116_Supplemental_Fig_S1.pdf]

Supplemental Fig S1

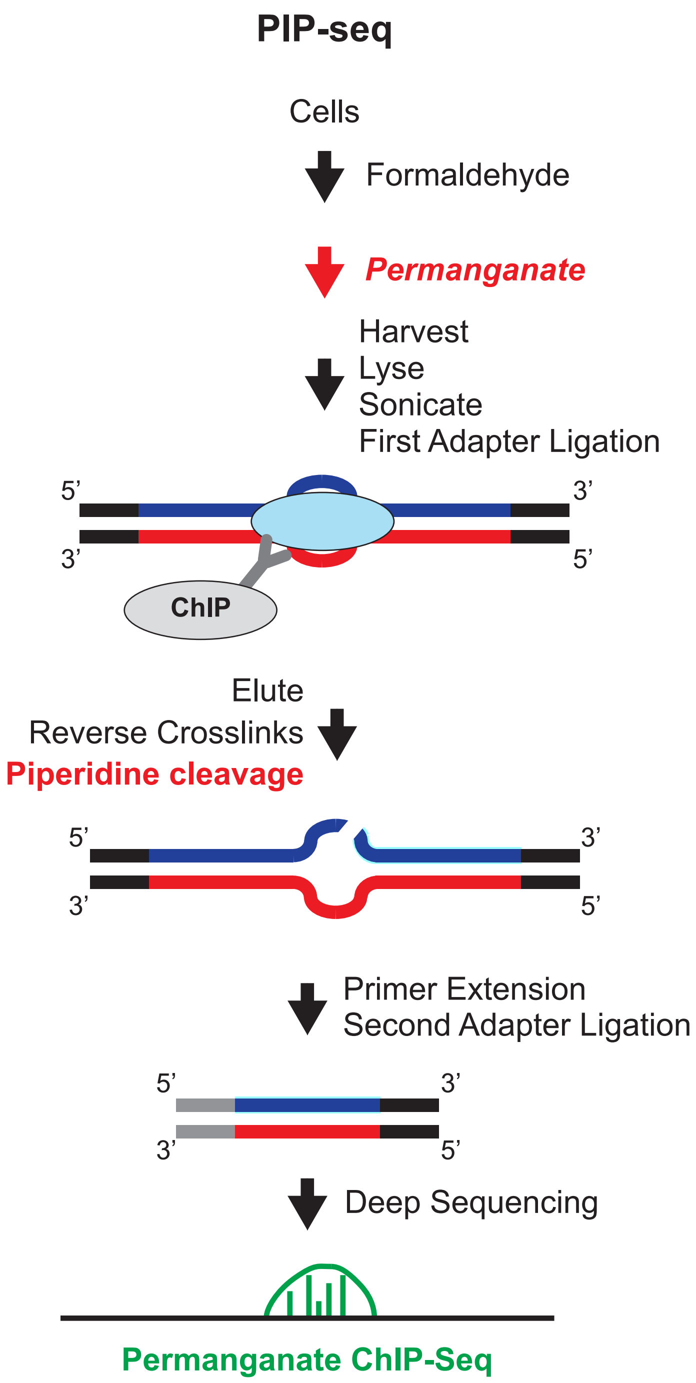

Supplemental Fig S1. PIP-seq protocol schema. Shown is an illustration of the PIP-seq assay protocol as previously described (Li et al. 2013). In short, cells are cross-linked with formaldehyde and treated with potassium permanganate. They are then lysed and sonicated to fragment sizes between 200-500 bp. An Illumina-compatible adapter is then ligated to the ends after end-repair and A-tailing. The sample is then eluted from the antibody and crosslinks arereversed. Piperidine is then used to cleave oxidized thymines. Strands are denatured and a primer is annealed to the adapters, followed by second-strand synthesis (primer extension). A second adapter is subsequently ligated to the piperidine-cleaved end, via A-tailing. The resulting library is then quantified and sequenced.
