# Supplementary material for: Genome-wide uniformity of human ‘open’ pre-initiation complexes
Source: Genome Res. 2017 Jan;27(1):15–26. doi: 10.1101/gr.210955.116 (PMC5204339; doi:10.1101/gr.210955.116)
Supplement: Supplemental Material [file supp_gr.210955.116_Supplemental_Fig_S2.pdf]

Supplemental Fig S2

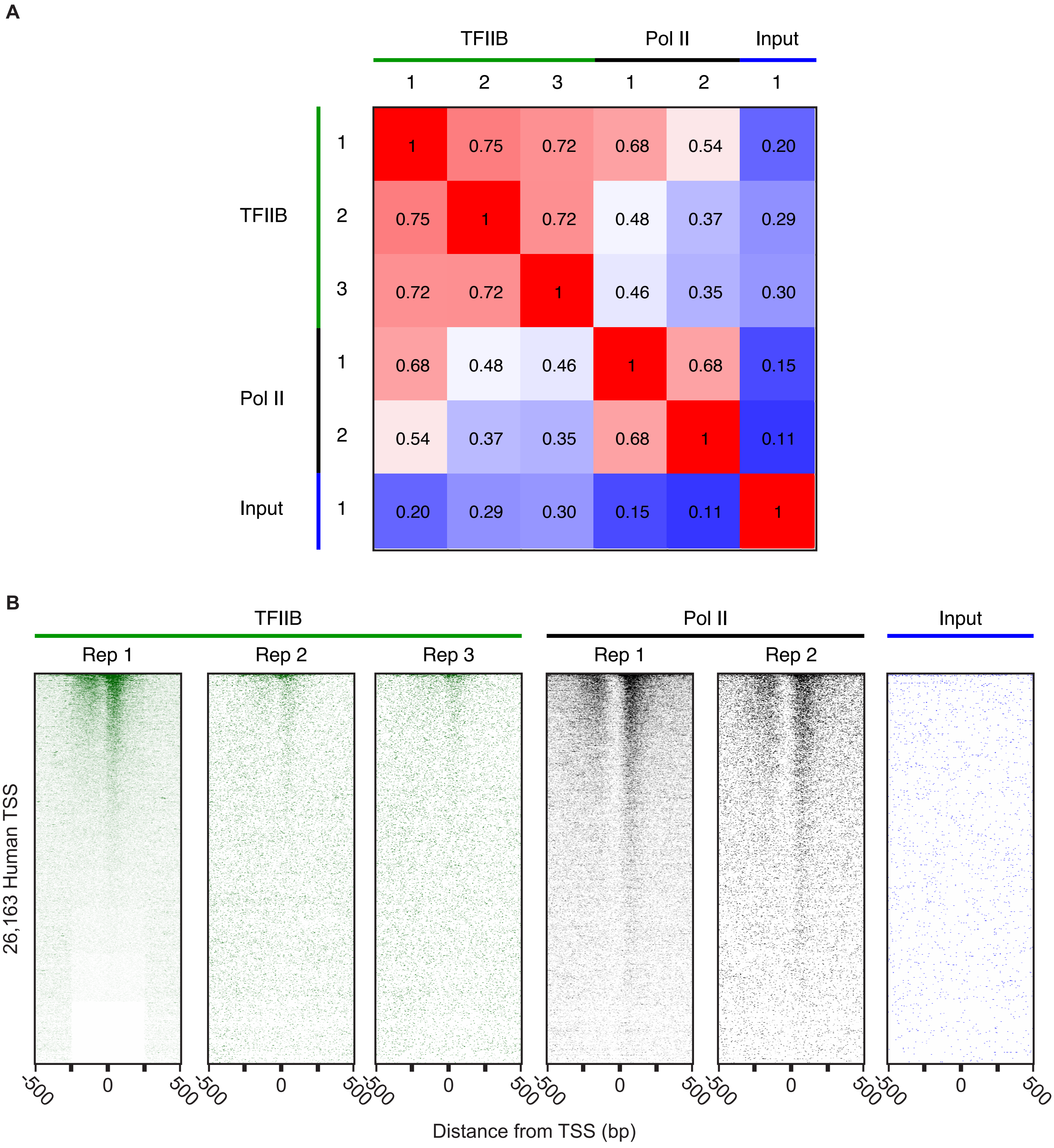

Supplemental Figure S2. Replicate correlation validation. (A) Pearson correlation matrix of the total tag count of TFIIB Rep 1, Rep 2, Rep 3, Pol II Rep 1, Rep 2, and Input in a 500 bp window centered at annotated human TSSs (N=26,163) (B) Heatmaps of all PIP-seq replicates in a 1kb window aligned relative to human TSSs (N=26,163) and sorted by total tag count of TFIIB replicate 1 in 500 bp window centered on TSS.
