# Supplementary material for: Genome-wide uniformity of human ‘open’ pre-initiation complexes
Source: Genome Res. 2017 Jan;27(1):15–26. doi: 10.1101/gr.210955.116 (PMC5204339; doi:10.1101/gr.210955.116)
Supplement: Supplemental Material [file supp_gr.210955.116_Supplemental_Fig_S3.pdf]

Supplemental Fig S3

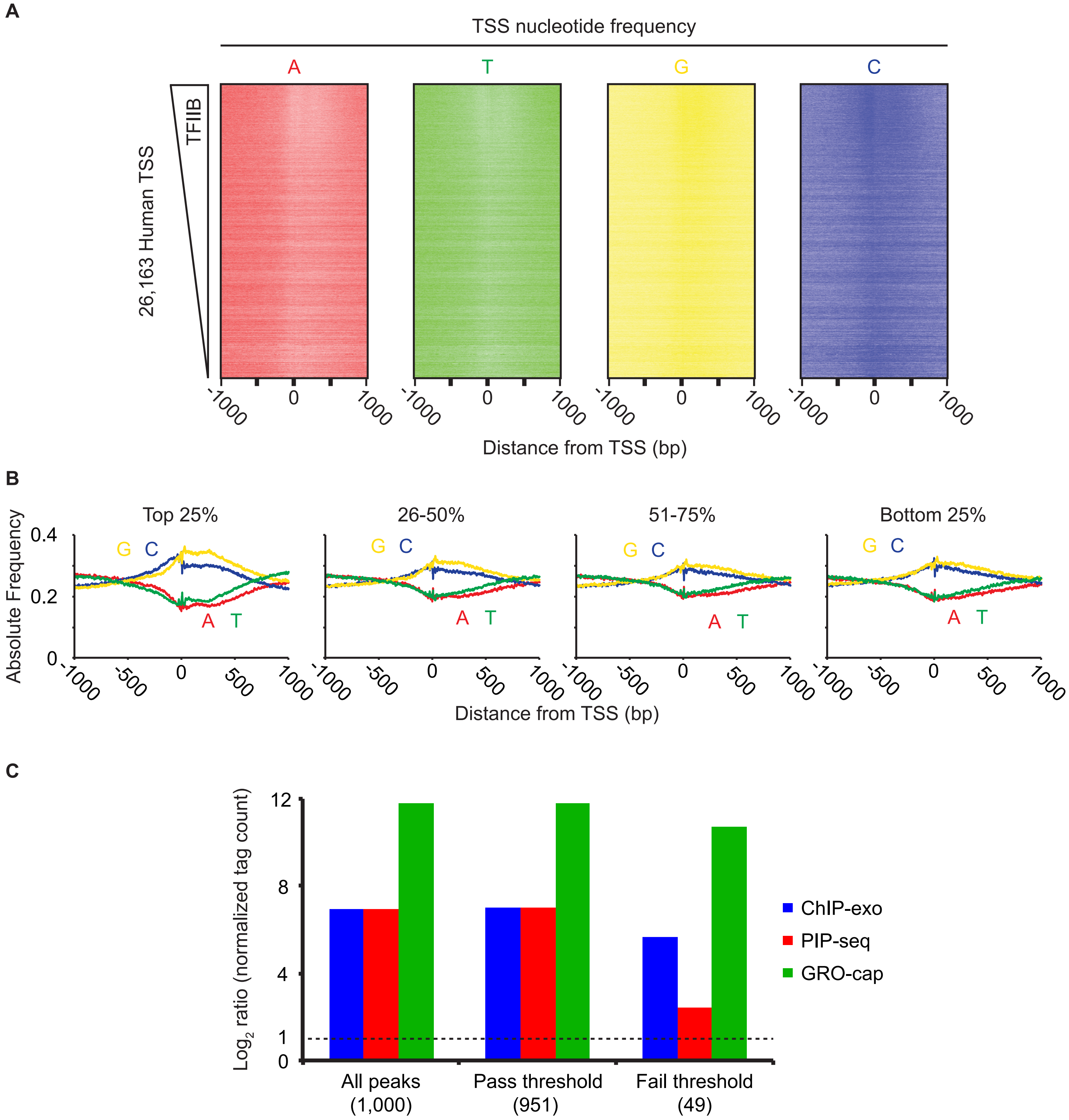

Supplemental Figure S3. Nucleotide frequencies surrounding annotated human TSS. (A) Heatmaps of nucleotide frequency within 1000 bp of annotated TSSs (N=26,163). Plots are sorted by TFIIB PIP-seq tag counts located within 250 bp of each TSS. Only those tag 5' ends that mapped just 3' to a 'T' were counted, when sorting. (B) Composite plots from panel A, separated out into quartiles of TFIIB PIP-seq peak tag counts. (C) Log_2 ratio of the average called peak score over the average random peak score for TFIIB ChIP-exo, TFIIB PIP-seq, and GRO-cap
