# Supplementary material for: Genome-wide uniformity of human ‘open’ pre-initiation complexes
Source: Genome Res. 2017 Jan;27(1):15–26. doi: 10.1101/gr.210955.116 (PMC5204339; doi:10.1101/gr.210955.116)
Supplement: Supplemental Material [file supp_gr.210955.116_Supplemental_Fig_S4.pdf]

Supplemental Fig S4

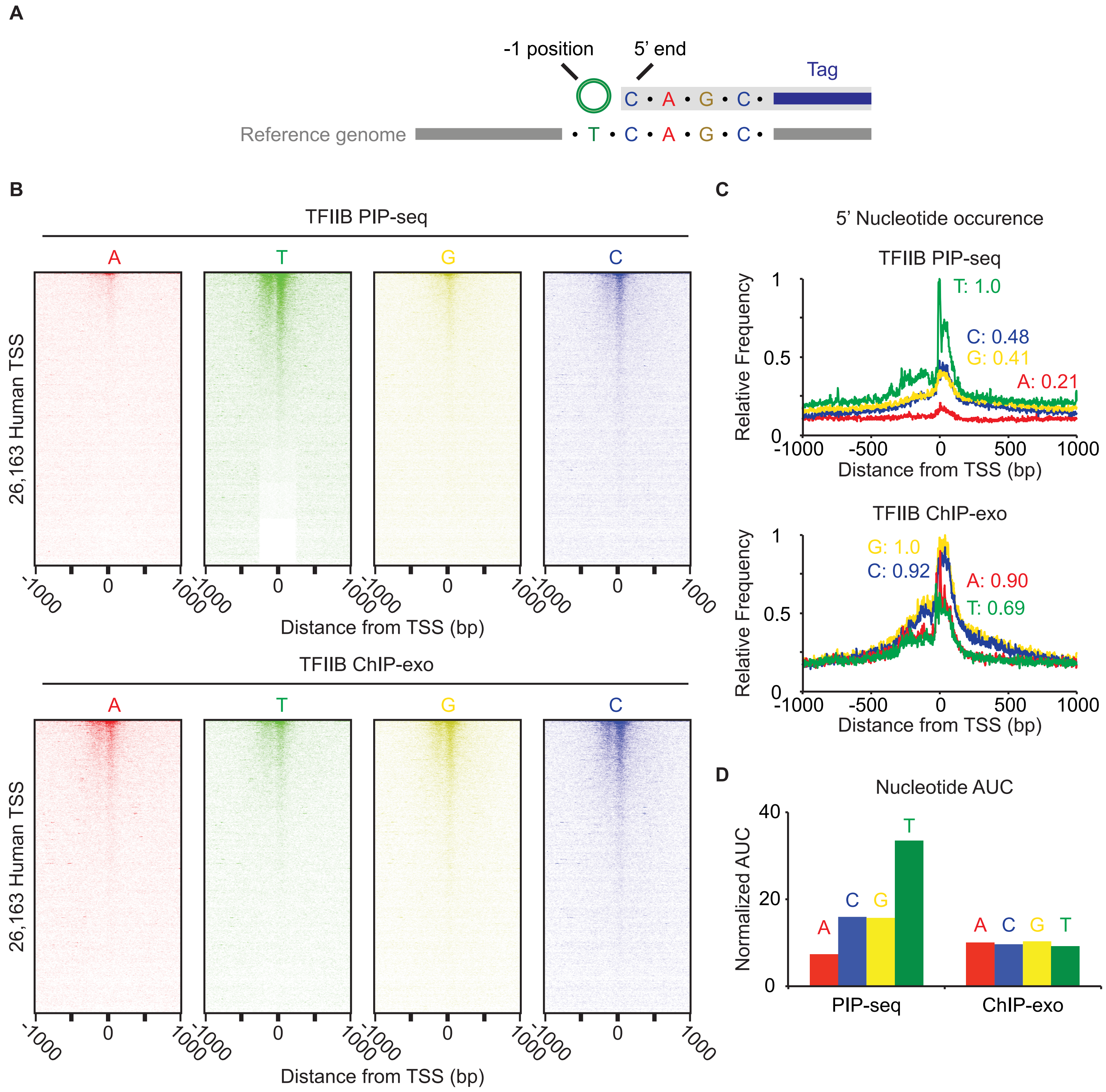

Supplemental Figure S4. TFIIB PIP-seq validation. (A) Schematic showing how reads were filtered by the specific -1 nucleotide 5’ to the aligned sequence read (B) Heatmap distribution of TFIIB PIP-seq (top) or ChIP-exo (bottom) tag 5’ ends relative to all annotated human TSSs, and separated out by the type of nucleotide (’A’, ‘T’, ‘G’, ‘C’) occurring 1 nucleotide upstream from the 5’ end (i.e., “-1” position). Plots are sorted by TFIIB PIP-seq tag counts (that also have a -1 ’T’) within 250 bp of each TSS. (C) Composite (average) of panel A. Relative peak height values (mode) for each composite are reported. (D) Area under the curve (AUC) from panel b normalized by the local nucleotide content, calculated as Σ(Avg reads ±250 bp window around TSS) / (Avg nucleotide frequency ±250 bp window around TSS)
