# Supplementary material for: Genome-wide uniformity of human ‘open’ pre-initiation complexes
Source: Genome Res. 2017 Jan;27(1):15–26. doi: 10.1101/gr.210955.116 (PMC5204339; doi:10.1101/gr.210955.116)
Supplement: Supplemental Material [file supp_gr.210955.116_Supplemental_Fig_S5.pdf]

Supplemental Fig S5

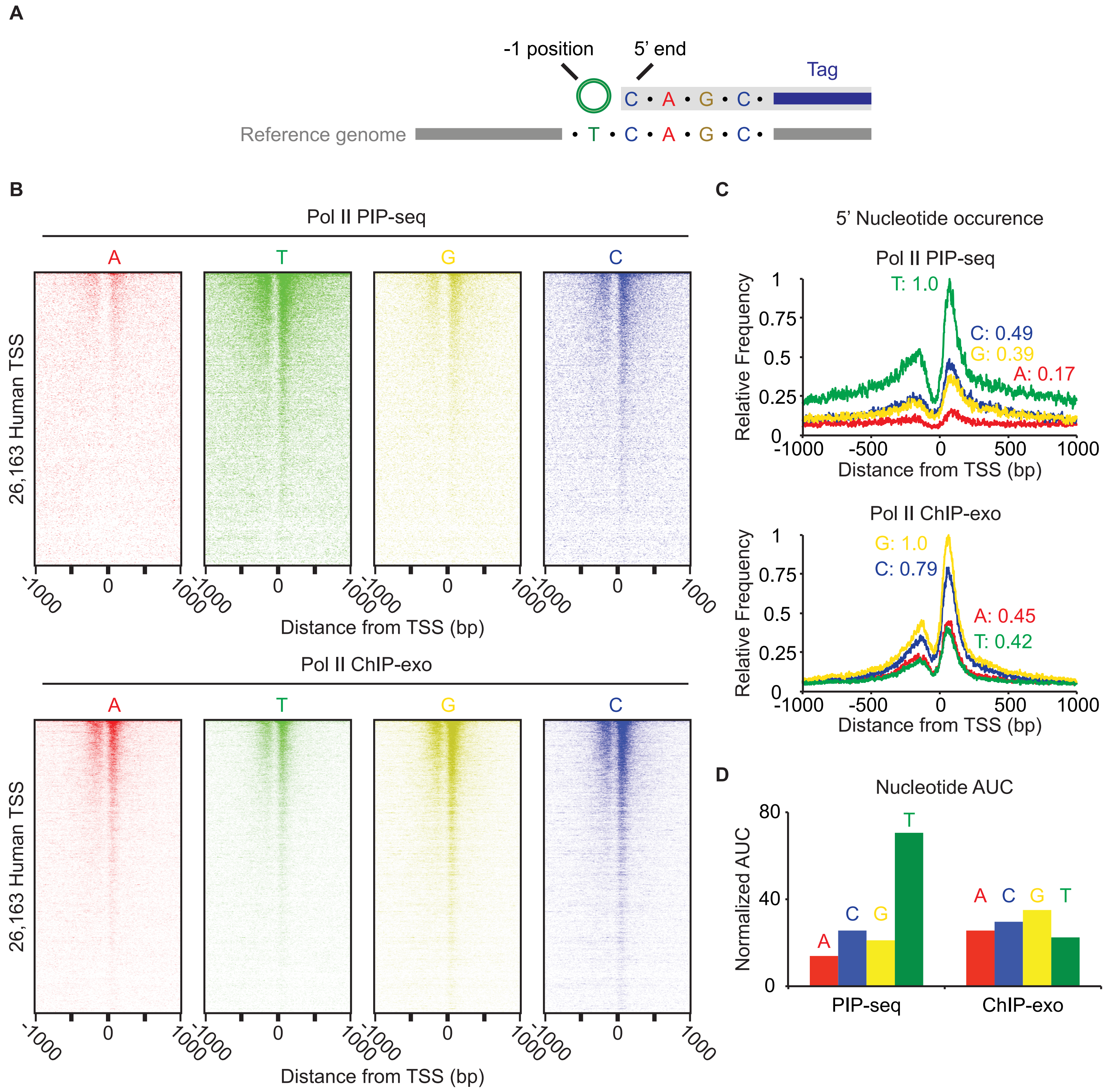

Supplemental Figure S5. Pol II PIP-seq validation. (A-D) Same as Supplemental Figure 3, except the analysis was on Pol II instead of TFIIB.
