# Supplementary material for: Genome-wide uniformity of human ‘open’ pre-initiation complexes
Source: Genome Res. 2017 Jan;27(1):15–26. doi: 10.1101/gr.210955.116 (PMC5204339; doi:10.1101/gr.210955.116)
Supplement: Supplemental Material [file supp_gr.210955.116_Supplemental_Fig_S6.pdf]

Supplemental Fig S6

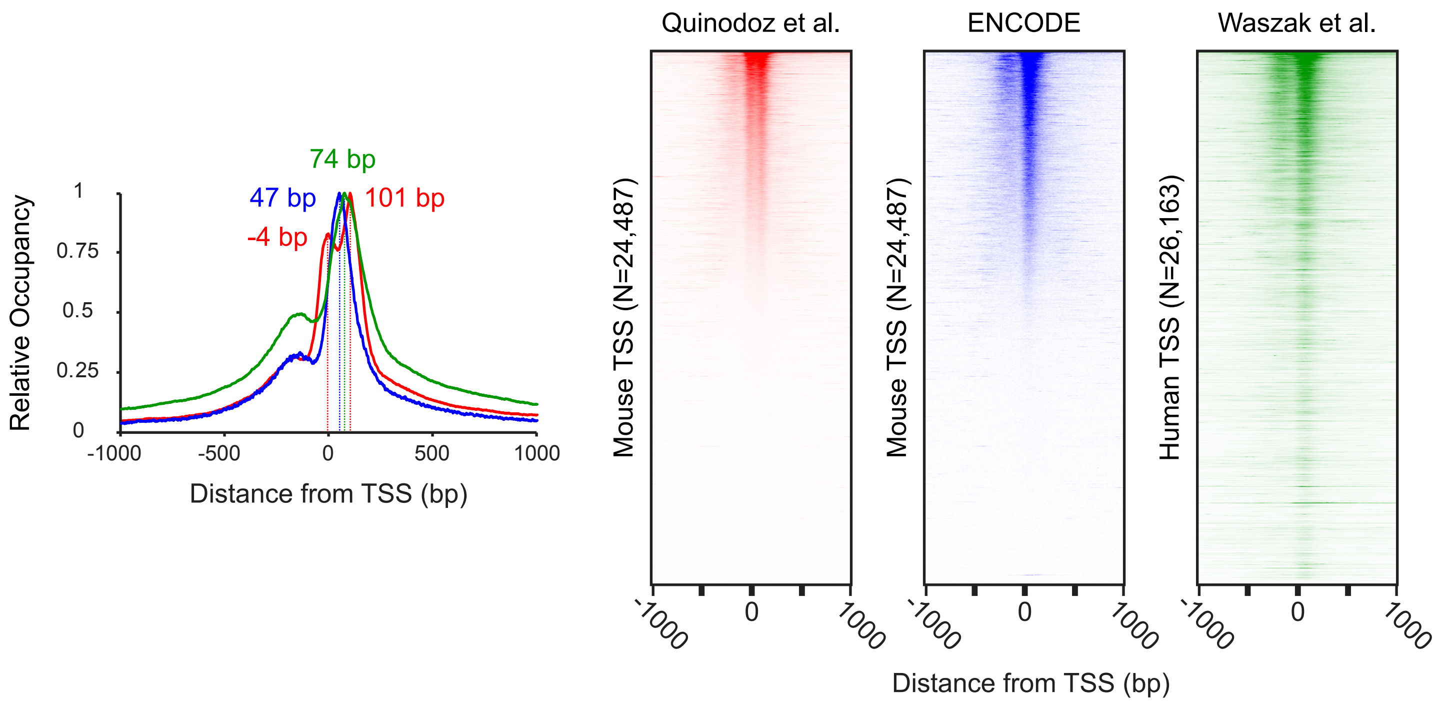

Supplemental Figure S6. Pol II ChIP-seq Tag Distribution. Composite plot and heatmaps of Pol II ChIP-seq 5' ends for Quinodoz et al. [GSE58443] (red), ENCODE [ENCFF001LNH] (blue), and Waszak et al. [NA06985] (green) aligned to each organisms' TSSs (mm10 and hg19). Local peak modes are noted in their respective colors. Pol II ChIP-seq was performed by Quinodoz et al. in mouse liver using Pol II antibody sc-67318, by ENCODE in mouse liver using sc-899, and by Waszak et al. in human leukocyte using sc-67318. 5' tag end shifts for Quinodoz et al., ENCODE, and Waszak et al. were respectively 75 bp, 40 bp, and 45bp. The resolution of ChIP-seq is based on fragment sonication (100-200bp) and therefore lacks the resolution needed to separate PICs and PCs. Deeper sequencing depth was reported in Quinodoz et al. as the reason for higher resolution, however there is no biochemical rationale to support that statement. Additionally, the sequencing depth for Quinodoz et al. and Waszak et al. was 177,277,241 and 126,801,813 uniquely aligned reads respectively and there is no evidence for PIC and PC seperation in Waszak et al.. Altogether, Quinodoz et al. is inconsistent with the vast majority of other data on Pol II PC location (including reported PC location). We therefore conclude the Quinodoz et al. data cannnot resolve PICs and PCs.
