# Supplementary material for: Genome-wide uniformity of human ‘open’ pre-initiation complexes
Source: Genome Res. 2017 Jan;27(1):15–26. doi: 10.1101/gr.210955.116 (PMC5204339; doi:10.1101/gr.210955.116)
Supplement: Supplemental Material [file supp_gr.210955.116_Supplemental_Fig_S7.pdf]

Supplemental Fig 7

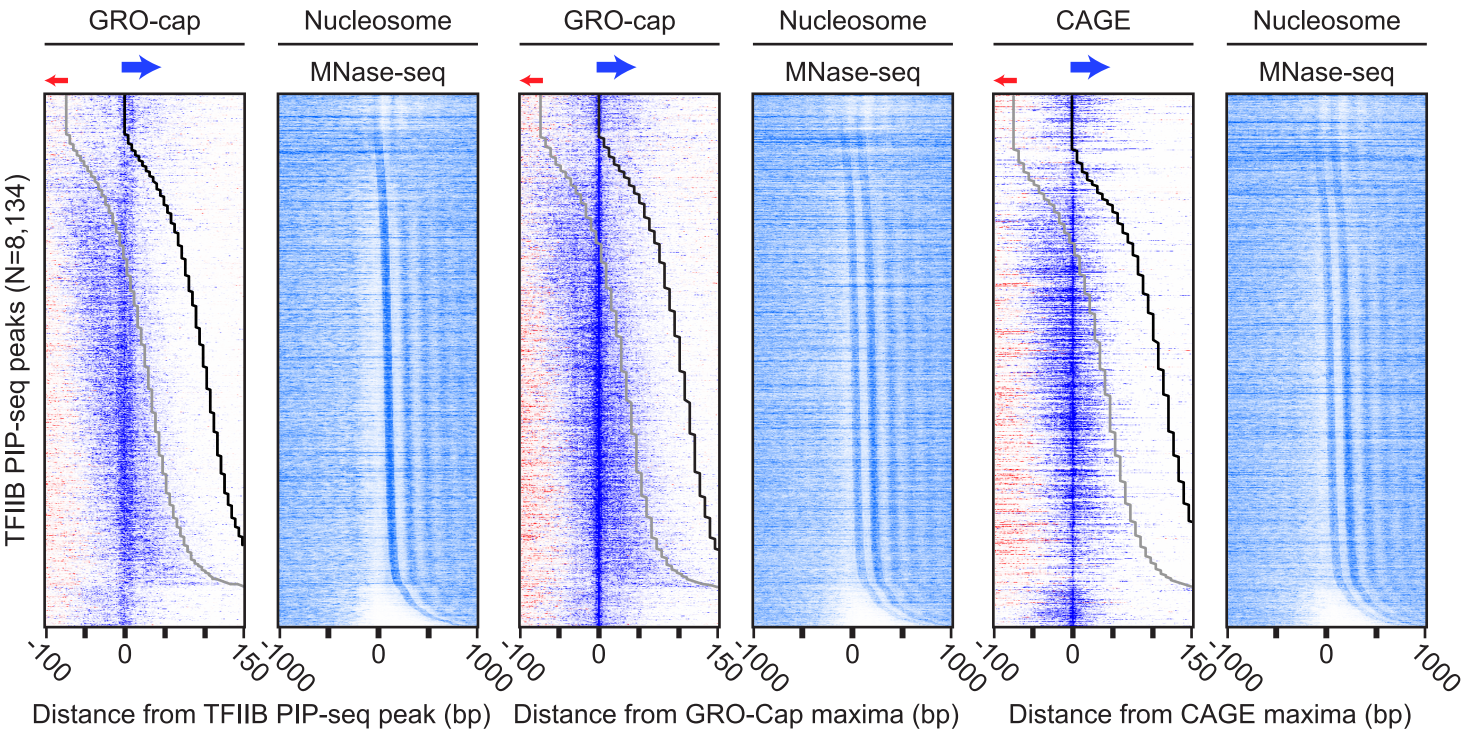

Supplemental Figure 7. Dispersion validation. Heatmaps of GRO-cap and CAGE with three distinct reference points: TFIIB PIP-seq peak, GRO-Cap local maxima, and CAGE local maxima. All heatmaps were independently sorted with respect to closest downstream +1 nucleosome determined as previously described.
