# Supplementary material for: Genome-wide uniformity of human ‘open’ pre-initiation complexes
Source: Genome Res. 2017 Jan;27(1):15–26. doi: 10.1101/gr.210955.116 (PMC5204339; doi:10.1101/gr.210955.116)
Supplement: Supplemental Material [file supp_gr.210955.116_Supplemental_Fig_S8.pdf]

Supplemental Fig S8

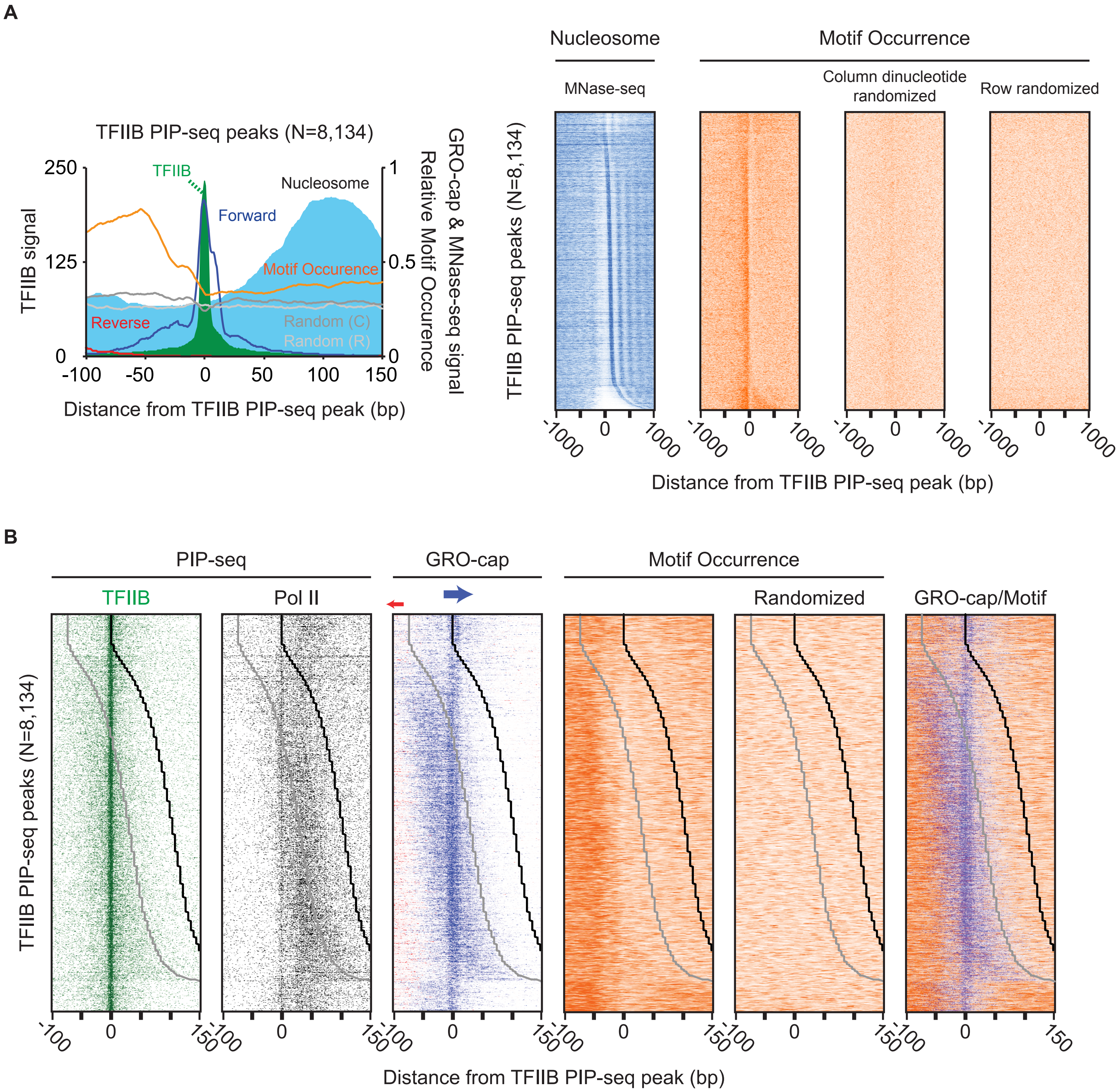

Supplemental Figure S8. Motif enrichment relative to PIC. (A) Composite plots and selected heatmaps of TFIIB PIP-seq, GRO-cap, MNase-seq, and the enriched motif occurrence count count relative to called TFIIB PIP-seq peaks. JASPAR 2016 vertebrate motifs were scanned using FIMO in a 2 kb window relative to TFIIB PIP-seq peaks. Control data was generated by randomizing either the dinucleotide frequency down the column to control for relative dinucleotide positioning or the sequence across the entire row from each 2 kb window to control for total nucleotide composition. Called motifs were aligned relative to called peaks and sorted by distance of TFIIB PIP-seq peak to called +1 nucleosome. The top five most common enriched motifs were: MA0528.1, MA0516.1, MA0079.3, MA0162.2, and MA0599.1. (B) Heatmaps of PIP-seq data displaying the overlap of the motif occurrence and the GRO-cap data. The black line is the location of the called +1 nucleosome dyad and the grey line is exactly 73 bp upstream representing the upper edge of the nucleosome.
