# Supplementary material for: Genome-wide uniformity of human ‘open’ pre-initiation complexes
Source: Genome Res. 2017 Jan;27(1):15–26. doi: 10.1101/gr.210955.116 (PMC5204339; doi:10.1101/gr.210955.116)
Supplement: Supplemental Material [file supp_gr.210955.116_Supplemental_Fig_S9.pdf]

Supplemental Fig S9

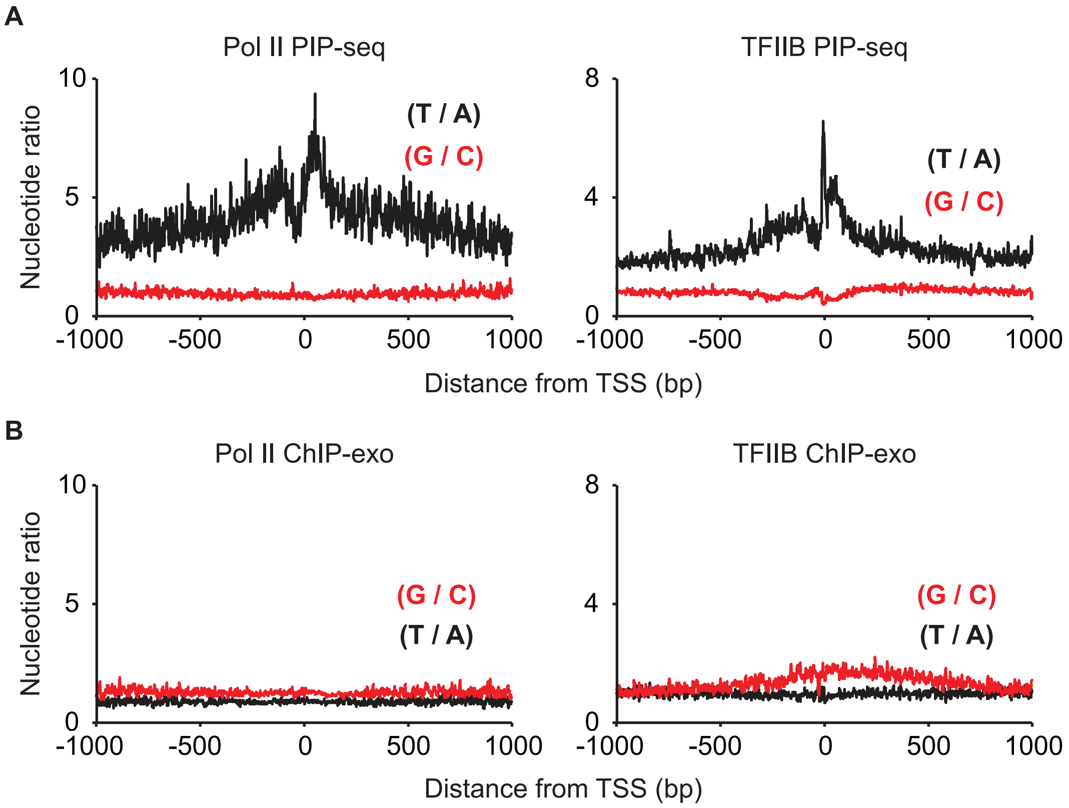

Supplemental Figure S9. PIP-seq strand normalization validation. (A) Pol II and TFIIB PIP-seq -1 'T' tag counts were aligned relative to annotated human TSSs (N=26,163), smoothed with a 7 bp sliding window average, and then locally normalized (divided) by -1 'A' tag counts treated similarly (T/A) so as to report on the relative T-reactivity relative to the equally occurring "A" nucleotide, as a readout of single-stranded DNA. Similar control plots were made for 'G' and 'C' tags (G/C). (B) Pol II and TFIIB ChIP-exo -1 'N' tags were normalized similarly as panel A.
