# Supplementary material for: Genome-wide uniformity of human ‘open’ pre-initiation complexes
Source: Genome Res. 2017 Jan;27(1):15–26. doi: 10.1101/gr.210955.116 (PMC5204339; doi:10.1101/gr.210955.116)
Supplement: Supplemental Material [file supp_gr.210955.116_Supplemental_Fig_S10.pdf]

# Supplemental Fig S10

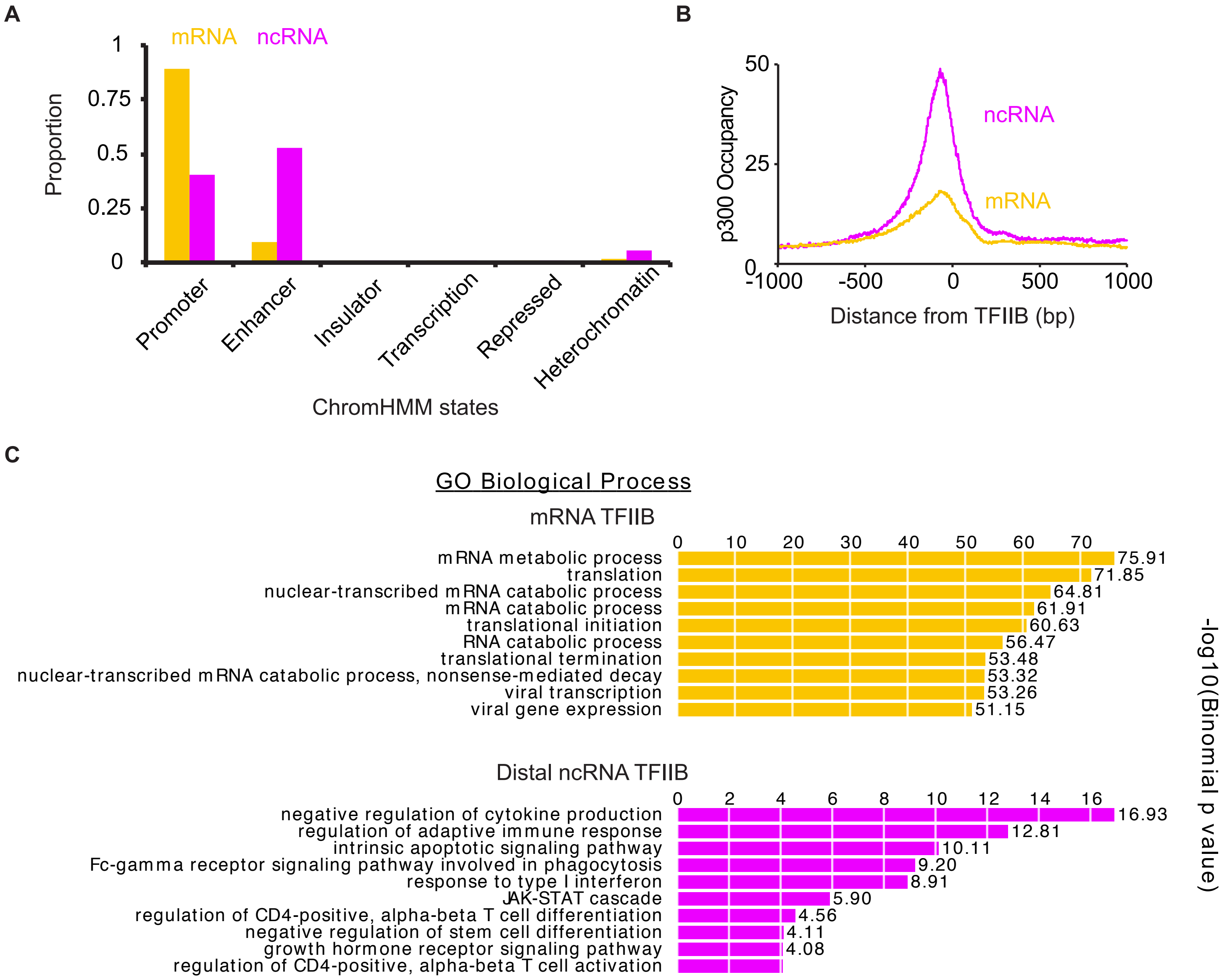


**Supplemental Figure S10. ncRNA and mRNA TFIIB meta-analysis.** (A) Proportion of predicted regulatory functions based on chromatin state maps (ENCODE K562 ChromHMM) for mRNA and ncRNA TFIIB locations. (B) Composite plot of ENCODE K562 p300 ChIP-seq occupancy plotted relative to mRNA and ncRNA TFIIB PIP-seq peaks. (C) GO enrichment of closest annotated genes for mRNA and distal ncRNA TFIIB locations using GREAT enrichment.
