# Supplementary material for: Genome-wide uniformity of human ‘open’ pre-initiation complexes
Source: Genome Res. 2017 Jan;27(1):15–26. doi: 10.1101/gr.210955.116 (PMC5204339; doi:10.1101/gr.210955.116)
Supplement: Supplemental Material [file supp_gr.210955.116_Supplemental_Table_S1.pdf]

Supplementary Table S1: Sequencing Experiment Statistics

	Assay	Replicate	Total Tags	Total 'T' Tags
Pol II	PIP-seq	1	62,908,427	26,593,623
Pol II	PIP-seq	2	11,845,451	4,949,154
TFIIB	PIP-seq	1	43,354,508	15,486,376
TFIIB	PIP-seq	2	18,579,509	7,258,212
TFIIB	PIP-seq	3	12,444,571	4,809,101
Input	PIP-seq	1	4,203,956	1,309,290
Pol II	ChIP-exo	1	19,743,244	-
TFIIB	ChIP-exo	1	14,723,536	-
